# Seasonal Variability of the Airborne Eukaryotic Community Structure at a Coastal Site of the Central Mediterranean

**DOI:** 10.3390/toxins13080518

**Published:** 2021-07-24

**Authors:** Mattia Fragola, Maria Rita Perrone, Pietro Alifano, Adelfia Talà, Salvatore Romano

**Affiliations:** 1 Department of Mathematics and Physics, University of Salento, Via per Arnesano, 73100 Lecce, Italy; mattia.fragola@unisalento.it (M.F.); mariarita.perrone@unisalento.it (M.R.P.); 2 Department of Biological and Environmental Sciences and Technologies, University of Salento, Via Monteroni, 73100 Lecce, Italy; pietro.alifano@unisalento.it (P.A.); adelfia.tala@unisalento.it (A.T.)

**Keywords:** eukaryotic community structure, airborne PM10 samples, high-throughput sequencing, fungi genus seasonality, plant genus seasonality

## Abstract

The atmosphere represents an underexplored temporary habitat for airborne microbial communities such as eukaryotes, whose taxonomic structure changes across different locations and/or regions as a function of both survival conditions and sources. A preliminary dataset on the seasonal dependence of the airborne eukaryotic community biodiversity, detected in PM10 samples collected from July 2018 to June 2019 at a coastal site representative of the Central Mediterranean, is provided in this study. Viridiplantae and Fungi were the most abundant eukaryotic kingdoms. Streptophyta was the prevailing Viridiplantae phylum, whilst Ascomycota and Basidiomycota were the prevailing Fungi phyla. *Brassica* and *Panicum* were the most abundant Streptophyta genera in winter and summer, respectively, whereas *Olea* was the most abundant genus in spring and autumn. With regards to Fungi, *Botrytis* and *Colletotrichum* were the most abundant Ascomycota genera, reaching the highest abundance in spring and summer, respectively, while *Cryptococcus* and *Ustilago* were the most abundant Basidiomycota genera, and reached the highest abundance in winter and spring, respectively. The genus community structure in the PM10 samples varied day-by-day, and mainly along with the seasons. The impact of long-range transported air masses on the same structure was also proven. Nevertheless, rather few genera were significantly correlated with meteorological parameters and PM10 mass concentrations. The PCoA plots and non-parametric Spearman’s rank-order correlation coefficients showed that the strongest correlations generally occurred between parameters reaching high abundances/values in the same season or PM10 sample. Moreover, the screening of potential pathogenic fungi allowed us to detect seven potential pathogenic genera in our PM10 samples. We also found that, with the exception of *Panicum* and *Physcomitrella*, all of the most abundant and pervasive identified Streptophyta genera could serve as potential sources of aeroallergens in the studied area.

## 1. Introduction

The microbial contents of soil and aquatic environments have been extensively investigated, while the atmosphere remains an underexplored biosphere [1,2], even if it represents a temporary habitat for airborne microorganisms—such as prokaryotes and eukaryotes—and also for microbial fragments. The eukaryotic community constitutes a significant fraction of the atmospheric microbial content, in addition to prokaryotes, and aerobiological studies are required to assess their emission, transport, deposition, diversity, and impact on the environment and human health. The airborne microbiome is aerosolized from different terrestrial and aquatic ecosystems [3,4], and then carried by air masses across transcontinental distances before being precipitated by wet and dry deposition processes [5]. Once deposited, interactions between the microbial entities may be activated in the new environment, thus contributing to biogeochemical cycles [6]. Moreover, the deposited microbiome can promote the dissemination of human, animal, and plant diseases and pandemics [7]. Tuberculosis and Spanish flu are typical examples of pandemics caused by bacterial or viral species transmitted through aerosols [8], as well as the current COVID-19 pandemic—speaking of which, Bourdrel et al. [9] have recently provided a review on the impact of outdoor air pollution on COVID-19 studies. Generally, the atmosphere is considered a hostile environment for the survival of microorganisms due to the lack of nutrients, the strong solar radiation, the large variations to which meteorological parameters (e.g., temperature, relative humidity, wind speed) are subjected, and even the presence of toxic molecules [10]. However, several studies have observed the presence of diverse and metabolically active airborne microbial communities, since these are mainly loaded on atmospheric particles [10], and in addition have shown that the airborne microbiome structure tends to differ depending on locations and regions, as survival conditions and sources vary. Therefore, its characterization and fluctuations, along with different atmospheric factors and geographical characteristics, have great significance. The recent advances in biological molecular techniques and culture-independent approaches have encouraged studies aimed at the characterization of the microbial communities in the atmosphere. The microbial community structure has mainly been characterized using culture-based methods in the past, but it is widely known that culture media capture only a small fraction of the total environmental microorganisms and, consequently, underestimate the airborne microbial diversity [11,12]. The advent of molecular methods and sequencing techniques has yielded novel insight into the airborne microbial community structure. Abd Aziz et al. [13] applied 16S rRNA gene sequencing to investigate the bacterial and fungal communities associated with PM2.5 mass concentrations both below and above the Korean air quality standard (36 μg m^−3^). Song et al. [14] used high-throughput sequencing techniques, targeting the 16S rRNA of bacteria and the 18S rRNA genes of eukaryotes, so as to characterize airborne microorganisms across the United Kingdom and provide a review on airborne microbes that commonly originate from soil and water through liquid–air and soil–air interfaces. Furthermore, atmospheric factors regulating the airborne microbiome communities at the local and global levels can serve as microbial indicators of specific bioaerosol sources and seasonality, and need to be demonstrated in different environments. Ruiz-Gil et al. [15] summarized and discussed recent advances in the study of airborne bacterial communities in outdoor environments, and the possible factors influencing their abundance, diversity, and seasonal variation. They also underlined how a more in-depth knowledge of the atmospheric microbiome of a particular region or country can contribute to implement new investigations focused on microbial ecology and the design of efficient regulations and policies for environmental protection and public health. In addition, Calderón-Ezquerro et al. [16] used a metagenomics approach on bacterial 16S and fungal ITS2 rRNA gene regions to investigate bacterial and fungal communities, respectively, in the atmosphere of Mexico City, while the microbial communities in the tropical air ecosystem have recently been investigated through the amplification and sequencing of 16S rRNA genes for bacteria and 18S rRNA genes for Fungi and Plantae [1]. 

In this study, we used 18S rRNA gene metabarcoding to characterize the structure and seasonal variability of airborne fungi and Viridiplantae, which are the main components of the eukaryotic community at the study site. More specifically, the parallel assessment of the biodiversity of airborne Fungi and Plantae communities, in addition to their seasonal fluctuations, represent this paper’s main goals, in order to contribute to drawing up a dataset on the airborne eukaryotic community biodiversity at a coastal site representative of the Central Mediterranean basin (Figure S1). In fact, the monitoring area, because of its geographical location, is significantly affected by long-range transported air masses from the surrounding countries and the Mediterranean Sea itself, alongside local and regional sources of pollution and bioaerosols, as shown in previous studies [17,18]. Therefore, the likely impact of long-range transported air masses and local meteorological parameters on the eukaryotic community structure was also investigated. 

Romano et al. [4,18] have recently used the 16S rRNA gene metabarcoding approach to characterize the structure of the airborne bacterial community in PM10 samples at the monitoring site, and investigate its dependence on meteorology, seasons, PM10 chemical components, and long-range transported air masses. Moreover, a preliminary local database on the potential airborne human- and plant-pathogenic bacterial species in PM10 samples collected at the study site has recently been reported [19].

## 2. Results and Discussion

### 2.1. Mass Concentrations and Meteorological Parameters

Table S1 lists the monitoring days and sampling time of the 37 samples collected from July 2018 to June 2019. Mean values of PM10 mass concentrations, temperature (T), relative humidity (RH), atmospheric pressure (P), wind direction and speed (WD and WS, respectively), and cumulative rain (CR) during the sampling time are also reported in Table S1. Seasonal mean values and their corresponding standard deviations (SD) of PM10, T, RH, P, WD, WS, and CR, for winter (January, February, March), spring (April, May, June), summer (July, August, September), and autumn (October, November, December), are listed in Table 1. PM10 mass concentrations and pressure levels did not vary with seasons within ±1 SD, in accordance with previous results at the study site [18,20,21]. In contrast, T, RH, WD, WS, and CR were season-dependent. The mean T value, which was equal to 8.7 °C in winter, increased to 26.1 °C in summer, while the RH mean values varied from 65 to 57% from winter to spring. The CR reached 39.1 mm in winter and 0.0 mm in summer (Table 1). The prevailing wind direction was northwest in winter, summer, and autumn, while it was southeast in spring. The WS mean value decreased from winter to autumn. The mean meteorological parameter values of this study were satisfactorily consistent with those from previous studies [18,22]. Note that the PM10 mass concentration and meteorological parameters for the 37 analysed samples were not normally distributed, according to the *p*-value estimated by the one-sample Kolmogorov–Smirnov test (at the 5% significance level), as reported in Table S2. The abnormal data distribution was likely due to the few data taken into consideration for the study.

### 2.2. Eukaryotic Community Structure at the Kingdom Level

**Figure 1:**
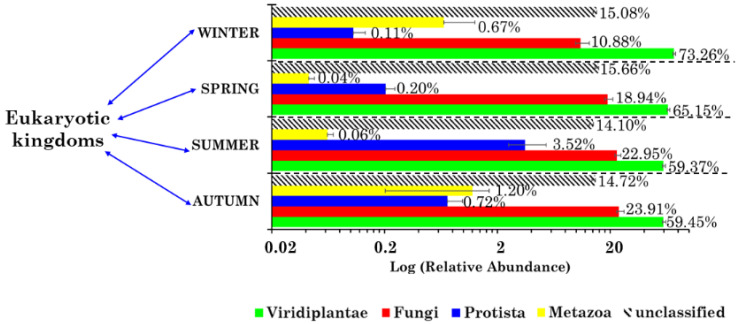
Mean percentage contribution (on a logarithmic scale) of the four eukaryotic kingdoms in each season: winter (January, February, March), spring (April, May, June), summer (July, August, September), and autumn (October, November, December). The percentage contributions due to the unclassified eukaryotic kingdoms are also provided. Error bars represent the standard error of the mean.

The 18S rRNA gene sequencing allowed the detection of four eukaryotic kingdoms (Viridiplantae, Fungi, Protista, and Metazoa) in the 37 samples collected from July 2018 to June 2019. Figure 1 shows their mean percentage contribution (on a logarithmic scale) in winter, spring, summer, and autumn, in addition to the corresponding contributions due to the unclassified eukaryotic kingdoms. The most abundant kingdom was Viridiplantae, whose percentage contribution decreased from winter (73.26%) to summer (59.37%). Fungi was the second most abundant kingdom and its percentage contribution increased from winter (10.88%) to autumn (23.91%). The percentage contribution of Protista varied within the 0.11 (winter)–3.52% (summer) range, while that due to Metazoa varied within the 0.04 (spring)–1.20% (autumn) range. Figure 1 clearly shows that the relative abundancies (RAs) of the identified eukaryotic kingdoms were season-dependent. More specifically, Viridiplantae reached its highest mean RA (73.26%) in winter—a value that was 6.7 times greater than the winter Fungi mean RA (10.88%)—while in autumn the Viridiplantae mean RA (59.45%) was 2.4 times greater than the corresponding Fungi mean RA (23.91%), which was on average the highest value reached by this kingdom. Gusareva et al. [1] also found that the plant-associated reads in the tropical air ecosystem collapsed at the level of Viridiplantae, according to 18S rRNA gene sequencing, but in this case the Fungi RA was on average more than 30 times greater than the mean Viridiplantae RA. Song et al. [14] also applied 18S rRNA gene sequencing to PM samples collected across the United Kingdom, reporting that Fungi and Plantae (Phragmoplastophyta phylum) contributed on average 48.3% and 37.4%, respectively. Findings from our research, which are partially contrasting with those from the above-cited studies, may be ascribed to the strong dependence of the eukaryotic kingdom emission sources on the geographical characteristics of the monitoring region/country, and its corresponding atmospheric factors. 

The taxonomic characterization of only the Viridiplantae and Fungi community structures is carried out in this paper, since these were the predominant and most pervasive eukaryotic kingdoms at the monitoring site, according to the 18S rRNA gene sequencing.

### 2.3. Viridiplantae and Fungi Community Structures at the Phylum Level

**Figure 2:**
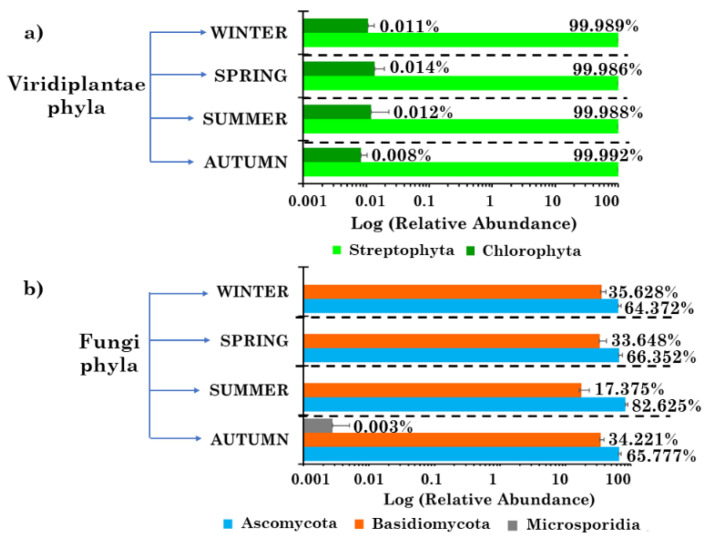
Seasonal mean percentage contribution (on a logarithmic scale) for the detected (**a**) Viridiplantae and (**b**) Fungi phyla in the 37 PM10 samples. Error bars represent the standard error of the mean.

Viridiplantae (i.e., green plants) are a clade of photosynthetic organisms that contain chlorophylls *a* and *b*, and produce and store their photosynthetic products inside a double-membrane-bounded chloroplast [23]. They are comprised of the Chlorophyta and the Streptophyta phyla. The Chlorophyta include most of the organisms typically referred to as “green algae”. The Streptophyta phylum comprises several other lineages that are also referred to as “green algae”, and the land plants, which include the liverworts, mosses, hornworts, lycopods, ferns, gymnosperms, and flowering plants [23]. Both phyla were detected in the PM10 samples of this study (Figure 2a), but the Streptophyta phylum was predominant, with a RA on average equal to 99.99%, besides being the most pervasive one. The Chlorophyta phylum contributed on average 0.01%. Banchi et al. [24] analysed the airborne plant taxonomic composition at the phylum level, by targeting the ITS2 gene with different primer combinations, across five locations in Northern and Central Italy. They also found that the Streptophyta phylum RA was far greater than that of the Chlorophyta. Núñez et al. [25] also targeted the ITS gene to characterize the plant diversity in the urban air of Madrid (Spain), and found that Chlorophyta and Bryophyta were the most abundant Viridiplantae phyla, likely because prevailing phyla are strongly dependent on the monitoring location/country and the corresponding atmospheric factors.

Within the Fungi kingdom, three phyla were detected in the analysed samples, i.e., Ascomycota, Basidiomycota and Microsporidia. Figure 2b shows the detected phylum RAs in winter, spring, summer, and autumn. Ascomycota represented the prevailing phylum in all seasons, and especially in summer, when its RA reached a value of 82.63%, while the Basidiomycota RA was equal to 17.37%. Microsporidia was only detected in two autumn samples (S22 and S32), and at very low RAs. The phylum Microsporidia is a large group of eukaryotic obligate intracellular parasites that can only complete their life cycle within an infected eukaryotic host cell [26]. Ascomycota and Basidiomycota are the two largest fungal phyla, widespread in many environments, including the atmosphere [14], and were further analysed in this study since they were the most abundant and pervasive ones in the collected PM10 samples. The predominance of Ascomycota in airborne particles, as compared to Basidiomycota Fungi, was reported in many studies performed worldwide [16,25,27]. Banchi et al. [28] found that fungal communities were richer in Basidiomycota than in Ascomycota—an opposite trend to that usually found in urban environments, where the pollution and lack of plant debris seem to favour the presence of some Ascomycota species.

### 2.4. Richness, Diversity, and Seasonal Dependence of Viridiplantae and Fungi Genera

**Figure 3:**
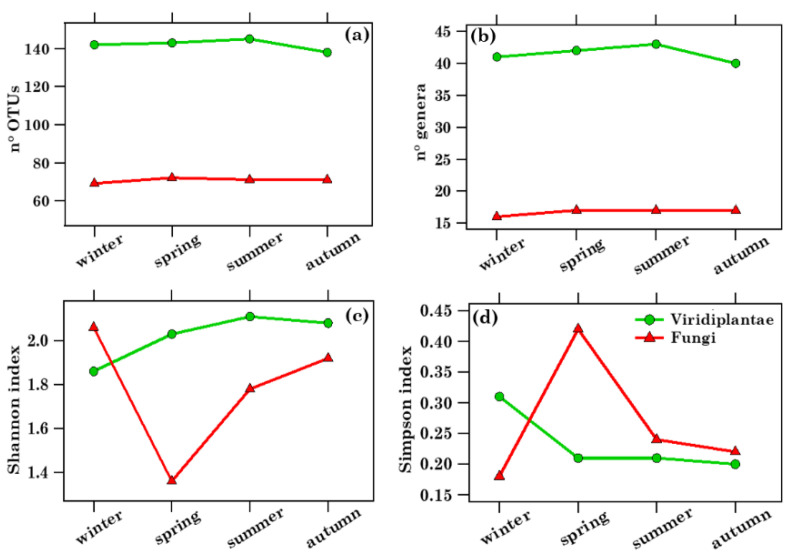
Comparison between the (**a**) number of OTUs, (**b**) number of genera, and (**c**) Shannon and (**d**) Simpson indices at the genus level for both Viridiplantae and Fungi across the 4 seasons: winter (January, February, March), spring (April, May, June), summer (July, August, September), and autumn (October, November, December). Viridiplantae data are displayed by green lines and dots, while Fungi data by red lines and triangles.

This study focused on the taxonomic characterization of the most abundant and pervasive Viridiplantae and Fungi phyla. Table S3 shows the heat map of the 46 airborne Viridiplantae Streptophyta genera detected in the 37 PM10 samples. The heat map for the 22 identified Fungi genera—consisting of 18 Ascomycota and 3 Basidiomycota genera, and 1 Microsporidia genus—is displayed in Table S4. Table 2 lists the number of the Viridiplantae OTUs and genera, and the Viridiplantae Shannon and Simpson index values at the genus level for the 37 analysed PM10 samples. Viridiplantae OTU and genus numbers varied within the 106–150 and 30–45 ranges, respectively, while Shannon and Simpson index values spanned the 0.91–2.43 and 0.12–0.69 ranges, respectively. The number of the Fungi OTUs and genera, along with the Fungi Shannon and Simpson index values at the genus level, are also listed in Table 2. The Fungi OTU and genus numbers varied within the 58–77 and 13–19 ranges, respectively, while the Fungi Shannon and Simpson index values spanned the 1.06–2.33 and 0.11–0.57 ranges, respectively. Therefore, Fungi OTUs and genus numbers were on average twice as small as the corresponding Viridiplantae parameters, likely because Viridiplantae were the prevailing kingdom over all seasons. Figure 3a–d shows the seasonal dependence of the mean OTU values and genus numbers, and of the Shannon and Simpson indices at the genus level for both Viridiplantae and Fungi, whose mean values ±1 standard deviation are also reported in Table S5. The mean values of the OTU and genus numbers barely varied with seasons. In contrast, the mean Shannon and Simpson index values were more affected by the seasons. The Shannon index *H* increases mostly with the species richness (total number of identified species), while the Simpson index *D*, which is a measure of dominance, increases as the diversity decreases. Their trend of variation is, therefore, opposite: in fact, Fungi richness was greatest in winter (*H* = 2.06) and smallest (*H* = 1.36) in spring, while their diversity was lowest (*D* = 0.18) in winter and largest in spring (*D* = 0.42). The Ascomycota and Basidiomycota genera, as well as the Streptophyta genera—whose RAs varied strongly with seasons, as shown in the following—can allow us to obtain a better understanding of the Shannon and Simpson indices’ variability with seasons. Note that Romano et al. [18] found at the study site that the OTU, phylum, and genus number mean values, concerning the Prokaryotic bacterial community, on average doubled from autumn/winter to spring/summer.

#### 2.4.1. Overview of Streptophyta Genera in the PM10 Samples

**Figure 4:**
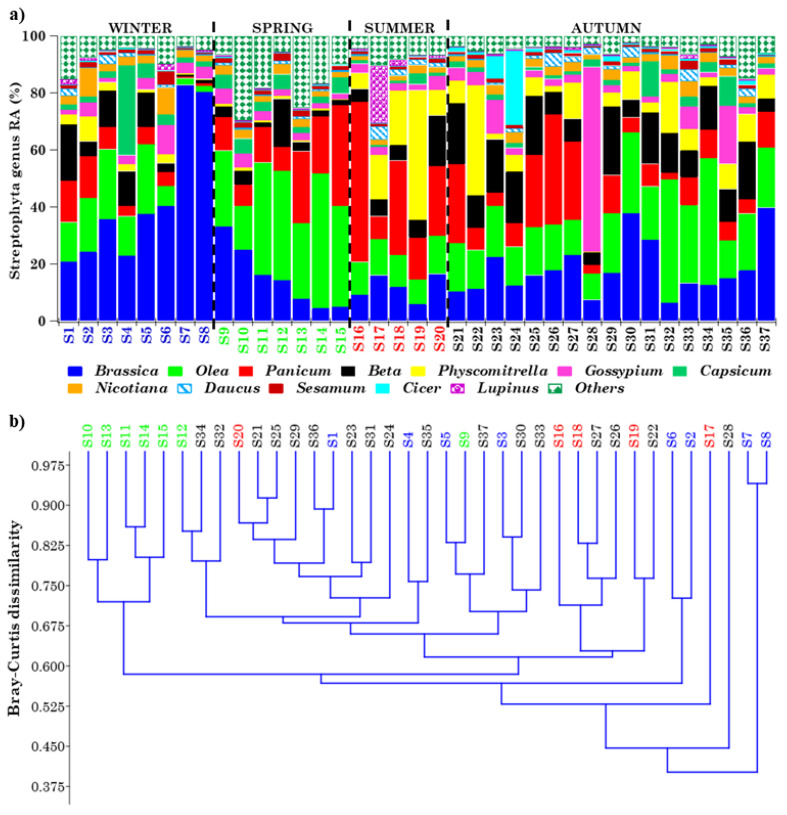
(**a**) Relative percentage contribution of the 12 most abundant and pervasive Streptophyta genera (≥1.17% mean within-sample relative abundance) in each of the 37 samples. The <1.17% mean within-sample relative abundance genera, in addition to the non-pervasive high-RA genera, are grouped as “*Others*”. (**b**) Bray–Curtis dissimilarity dendrogram highlighting the relatedness of the Streptophyta genera communities in the analysed samples: winter genera are in blue, spring genera in green, summer genera in red, and autumn genera in black.

**Figure 5:**
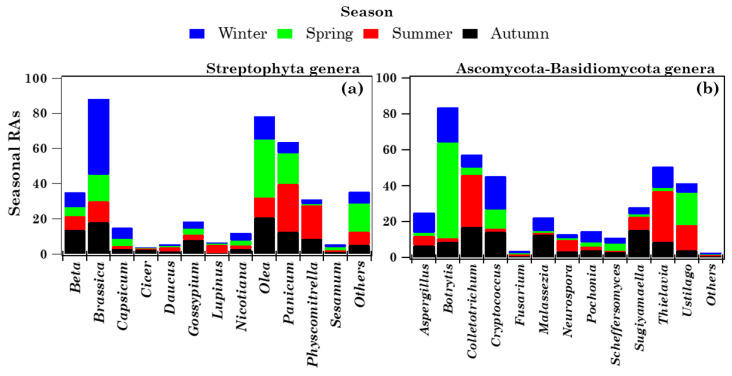
Stacked bar plots of the 12 most abundant and pervasive (**a**) Streptophyta (≥1.17% mean-within sample RA) and (**b**) Ascomycota/Basidiomycota (≥0.95% mean within-sample RA) genera across the four seasons. Each colour indicates a season, while the height of each colour bar represents the relative abundance of the corresponding genus in that season.

Forty-six airborne Viridiplantae Streptophyta genera were overall detected in the thirty-seven PM10 samples, as mentioned above (Table S3), but this study focused on the taxonomic characterization and seasonal dependence of the twelve most abundant and pervasive genera. Note that a genus is considered pervasive if it is detected in all of the samples except one. Figure 4a shows the RAs of the 12 most pervasive and abundant genera (mean within-sample RA ≥ 1.17%) in each of the detected samples, where “*Others*” represents the < 1.17% RA genera or high-RA non-pervasive ones. Apart from *Physcomitrella*, which were not detected in sample S28, all of the other genera were detected in all samples. The RA of the 12 most abundant genera, aside from varying from sample to sample, exhibited a strong seasonal dependence, as clearly shown in Figure 5a, where winter, spring, summer, and autumn samples are blue, green, red, and black coloured, respectively. The percentage contributions of the less abundant (<1.17% RA) and/or non-pervasive genera are indicated as “*Others*”, whose RA reached the highest value in spring. The mean percentage value (on a logarithmic scale) of the 12 most abundant and pervasive genera is also provided in Figure S2 for (a) winter, (b) spring, (c) summer, and (d) autumn. Error bars in Figure S2 represent the standard error of the mean. The Bray–Curtis dissimilarity dendrogram, based on the genus–RA Bray–Curtis matrix shown in Table S6, is displayed in Figure 4b. It shows the relatedness between the 37 samples, which are marked by different colours to clearly identify the sampling season. Except for the cluster made up of the spring samples S10, S11, S13, S14, and S15, most of the main clusters in Figure 4b consisted of samples collected in different seasons, possibly because samples sharing similar genus structures/ RAs were collected in different seasons, as a quick look at Figure 4a also reveals. The allergy-inducing *Brassica* [28] and *Panicum* were the most abundant genera in winter (43.13%) and summer (27.35%), respectively, while *Olea* was the most abundant genus in spring (32.71%) and autumn (20.82%). The few samples of Figure 4a in which the RA of a single genus is prevailing (> 60%), and is far above its mean value, may deserve special attention, since they could help to determine the environmental conditions and/or the atmospheric factors likely responsible for atypical-RA genera, and in this way contribute to a better understanding of the genus community seasonality. Figure 4a shows that *Brassica* reached RAs of 82.86 and 80.47% in samples S7 and S8, respectively, which were collected on February 21 and 28, 2019, respectively, i.e., in the winter season, when the genus *Brassica* RA was the highest (43.13%). Consequently, the Shannon and Simpson indices (Table 2) reached some of the highest (*H* = 0.91 and *H* = 1.04) and lowest (*D* = 0.69 and *D* = 0.65) values in S7 and S8, respectively, and *BC_7,8_* and all *BC_7,j_* and *BC_8,j_* indices were characterized by values ≥ 0.45, because of the high dissimilarity between the corresponding samples. Table S1 shows that PM10 mass concentrations reached the highest values on February 21 and 28, 2019 (47 and 44 μg m^−3^, respectively), and likely contributed to the rather high *Brassica* RAs detected in S7 and S8. The four-day HYSPLIT back trajectories show that the air masses that reached the study site at 12:00 UTC on February 21 (Figure S3a) and 28 (Figure S3b), 2019 crossed Eastern European countries before arriving at the study site, and likely contributed to the increase in the genus *Brassica* RA, since *Brassica* is native to Europe, and is especially common in the Mediterranean region. *Gossypium* reached an RA of 65.13%, which is far above its mean value (Figure 4a), in sample S28, collected on 21–22 November 2018; it grows mainly in tropical and subtropical warm, humid climates, and has an important role in the world of agriculture and trade. HYSPLIT back trajectories show that air masses from northern Africa and the Central Mediterranean sea were advected at the study site on 21–22 November 2018 (Figure S4a and S4b). Moreover, Figure S5 shows that the Mediterranean basin was affected by desert dust on those days, according to the dust load map from the BSC-DREAM8b model. Therefore, the advection of *Gossypium* from the northern African regions, where it is grown, likely contributed to its atypical RA as monitored on 21–22 November 2018. We believe that the above-reported case studies could be considered typical examples of the presumable impact of long-range transported air masses on the Streptophyta genus community structure. Banchi et al. [24,28] also found that *Brassica* was one of the most abundant genera in all seasons across five localities in Northern and Central Italy, and that it was predominant in summer and autumn, in reasonable accordance with the results of this study. Except for *Brassica*, none of the other most abundant and pervasive genera of this study were common to the ones detected by Banchi et al. [24]. In contrast, the less abundant (RAs < 1.17%) *Cucumis*, *Daucus*, and *Primus* (Table S3) were also detected by Banchi et al. [24] across all of the five monitored locations in Northern and Central Italy. Note that *Daucus* and *Primus* were identified in all samples at the monitoring site, whereas *Cucumis* was not in 6 out of the 37 samples. The main contributors to the airborne plant community may be represented by the ornamental species grown in urban areas and/or wild plants from natural areas, which are expected to be site- and season-dependent [25]. On the contrary, it is supposed that bacteria and fungi have on average a steady core of taxa, present in high abundance throughout the year.

#### 2.4.2. Overview of Ascomycota and Basidiomycota Genus Communities in PM10 Samples

**Figure 6:**
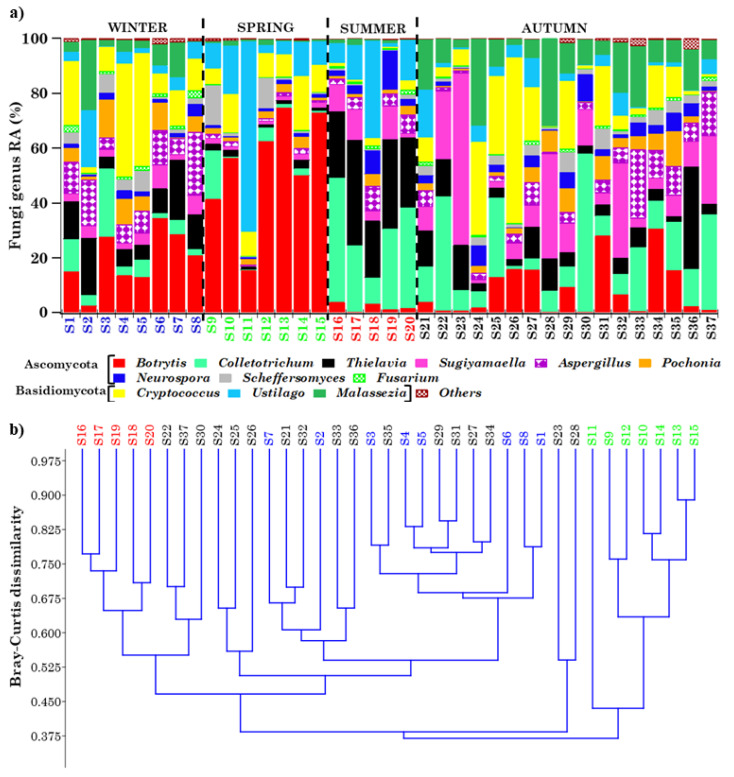
(**a**) Relative percentage contributions of the 12 most abundant and pervasive Fungi (Ascomycota/Basidiomycota) genera (≥0.95% mean within-sample relative abundance) in each of the 37 samples. The <0.95% mean within-sample relative abundance genera, in addition to the non-pervasive, high-RA ones, are grouped as “*Others*”. (**b**) Bray–Curtis dissimilarity dendrogram highlighting the relatedness of the genus-level Fungi communities in the analysed samples: winter genera are in blue, spring genera in green, summer genera in red, and autumn genera in black.

Figure 6a shows the RA of the 12 most pervasive and abundant (mean within-sample RA ≥ 0.95%) Fungi genera in the 37 samples, with the first 9 and the last 3 genera belonging to the Ascomycota and Basidiomycota phyla, respectively. The 12 selected most abundant genera were detected in all samples. Their strong seasonality is highlighted by Figure 5b, which displays the seasonal dependence of the 12 most abundant and pervasive Fungi genera RAs. “*Others*” represents the contribution of the less abundant (<0.95% RA) or high-RA, non-pervasive genera, which was distinctly scarce in every season, in contrast to the Streptophyta “*Others*” RAs displayed in Figure 4a. The mean percentage RA value (on a logarithmic scale) of the 12 most abundant and pervasive Fungi genera is provided in Figure S6 for (a) winter, (b) spring, (c) summer, and (d) autumn, where error bars represent the standard error of the mean. *Botrytis* and *Colletotrichum* were the most abundant Ascomycota Fungi, reaching the highest RAs in spring (53.35%) and summer (29.04%), respectively (Figure 5b and S6). Within the Basidiomycota phylum, *Cryptococcus* and *Ustilago* were the most abundant genera, since they reached the highest RAs, i.e., 18.52% and 17.89%, in winter and spring, respectively. The Bray–Curtis dissimilarity dendrogram, based on the genus–RA Bray–Curtis matrix (Table S7), is shown in Figure 6b. Most of the main clusters identified by the dendrogram were mainly made up of samples collected in the same season, as the clusters consisting of spring and summer samples. This last result might indicate that samples with a similar genus structure were mainly collected in the same season, in contrast to the results on Streptophyta (Figure 4b). In fact, a quick look at Figure 6a clearly reveals that the Fungi genus RAs in the samples varied more strongly with seasons, compared to the Streptophyta genera shown in Figure 4a. Figure 6a shows that a few genera reached atypical RAs in one or two samples; in particular, the Basidiomycota genus *Ustilago,* which reached an RA of 69.50% in sample S11, on average reached RAs smaller than 20% in most samples (Table S4). The 24-h sample S11 was collected on 9 May 2019. The four-day HYSPLIT back trajectories (Figure S7) indicate that the air masses likely crossed northern Africa and the Mediterranean Sea at very low altitudes, before reaching the study site at 12:00 UTC on 9 May 2019. Moreover, Figure S8 shows that the Mediterranean basin was affected by desert dust on that day, according to the dust load map from the BSC-DREAM8b model.

Therefore, the air masses advected at the monitoring area on May 9, 2019 may have contributed to the atypical RA of *Ustilago* in S11. The Shannon and Simpson indices of the Fungi genera reached the smallest (*H* = 1.06) and one of the greatest (*D* = 0.52) values in S11, and the *BC_11, j_* values were ≥ 0.53, because of the atypical genus structure of the sample. The Ascomycota *Sugiyamaella* reached an anomalous RA (62.55%) in the 48-h S23 sample collected on 17–18 October, 2018. Its RA was on average a few percent in winter, spring, and summer, but reached a mean RA equal to 15.33% in autumn. As a consequence, the Shannon and Simpson indices at the genus level reached one of the greatest (1.30) and smallest (0.43) values in S23, and the *BC_23, j_* indices were on average rather high because of the atypical genus structure of the sample. The PM10 mass concentration also reached one of the highest values (40 μg m^−3^) in S23 (Table 1). Air masses coming from Eastern Europe and Asia Minor reached the study site at 12:00 UTC on 17–18 October, 2018, according to the 4-day HYSPLIT back trajectories (Figure S9a,b), so they could have contributed both to the anomalous *Sugiyamaella* RA and to the increase in the PM10 mass concentration, whose value in S23 was far above the annual mean level. The *Sugiyamaella* genus has a worldwide distribution, and most of its species were originally found in Europe, North and South America and, more recently, in China. They were isolated either directly from wood-ingesting insects and insect frass, or from common insect habitats, such as rotting wood, forest soil, mushrooms, and peat [29]. In conclusion, it was shown that the analysis of samples with a prevailing (>60%) and atypical genus RA could help to infer the presumable impact of long-range transported air masses on the Ascomycota and Basidiomycota genera community structures, as found for the Streptophyta genera. When taking into account previous aerobiological research, Núñez et al. [25] also detected the Basidiomycota *Ustilago*, *Cryptococcus*, and *Malassezia* in the urban atmosphere of Madrid. They found that the most abundant genus was *Ustilago*, which reached the highest RA in spring, in accordance with our results. Moreover, they detected the Ascomycota *Fusarium* and *Aspergillus*, with mean RAs similar to the ones in this study. Du et al. [27] detected the genera *Fusarium* and *Aspergillus* in Beijing, with the latter being the prevailing genus, as in our study. The genera *Aspergillus*, *Ustilago*, and *Botrytis* were also detected in PM2.5 samples collected in Gwangju (South Korea) [13]. By contrast, none of the Fungi genera detected in a nine-month-long survey, across five locations in Northern and Central Italy [24], were common to the ones identified in our samples, apart from the less abundant *Candida* that they detected only in one site, i.e., in Umbria. In our study, *Candida* was detected in 31 out of the 37 analysed samples and, overall, it represented the 14th most abundant genus, reaching an average RA of about 0.14% (Table S4).

### 2.5. PCoA Analyses of Streptophyta and Ascomycota/Basidiomycota Genera in PM10 Samples

In addition to Bray–Curtis dissimilarity dendrograms, which were firstly used to highlight the relatedness between samples, the PCoA (principal coordinates analysis) technique was also applied to visualize gradients in the 37 investigated samples. PCoA represents a common exploratory analysis, allowing a graphical representation of the similarity/dissimilarity between objects [30], and its performance can be evaluated by the percentage of total variance explained by the first and second synthetic axes (components). 

**Figure 7:**
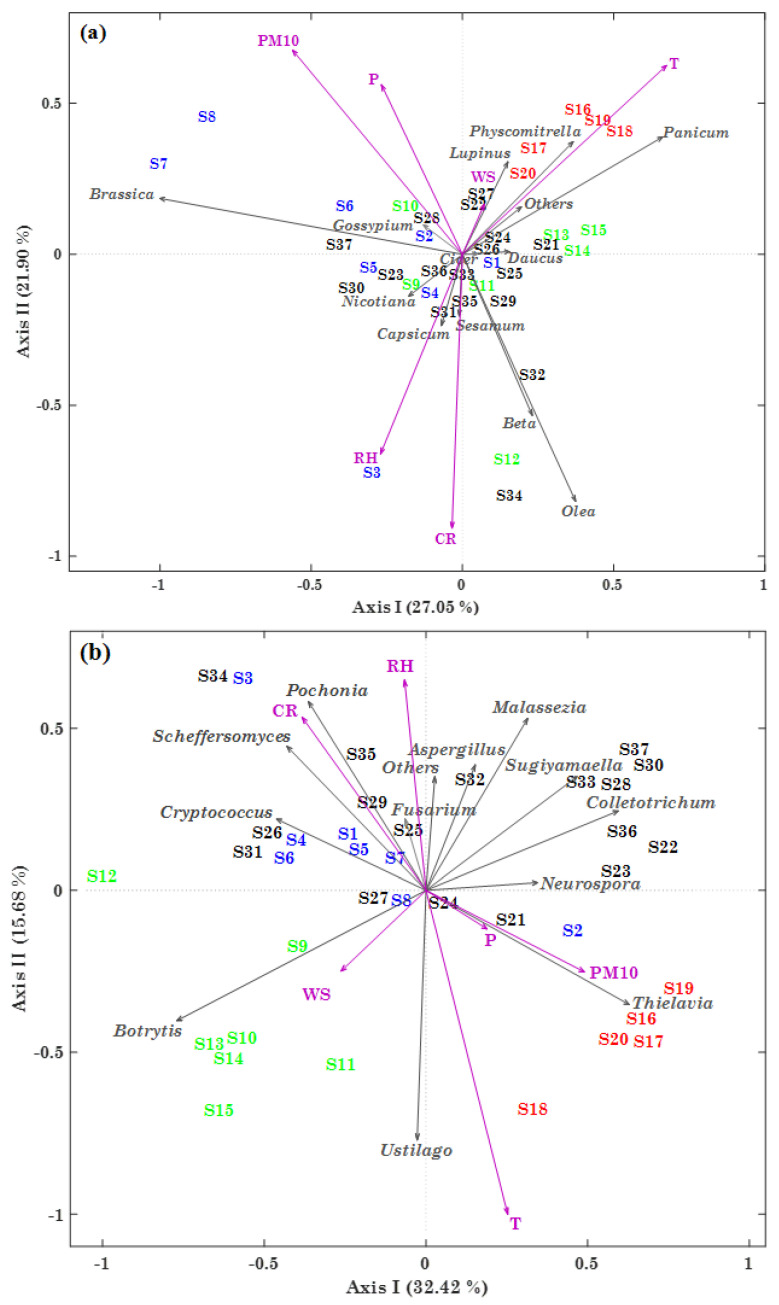
Two-dimensional principal coordinates analysis (PCoA) based on Bray–Curtis distances for the (**a**) 12 most abundant and pervasive Streptophyta and (**b**) Fungi genera RAs, meteorological parameters (except for WD), and PM10 mass concentrations in the 37 analysed samples. Winter samples (S1–S8) are coloured in blue, spring samples (S9–S15) in green, summer samples (S16–S20) in red, and autumn samples (S21–S37) in black. The percentages of the total variance explained by the first and second principal components are also indicated in the plot.

Figure 7a shows the two-dimensional PCoA ordination plot based on the *BC_i,j_* distances calculated from the 12 most abundant and pervasive Streptophyta genera RAs, meteorological parameters, and PM10 mass concentrations among the 37 samples. Winter (S1–S8), spring (S9–S15), summer (S16–S20), and autumn (S21–S37) samples are marked in blue, green, red, and black, respectively. The total variance percentages explained by the first and second axes were 27.05% and 21.90%, respectively. Analysing the correlation arrows depicted in Figure 7a, we were able to identify the Streptophyta genera, the meteorological parameters, and the PM10 concentrations characterized by the most significant correlations with sample ordinations. In particular, observe from Figure 7a that summer samples are located in the upper-right quadrant of the PCoA ordination plot, highlighting their different properties with respect to the Streptophyta genera community composition and the other analysed parameters in the other seasons. Considering the length of the correlation arrows in Figure 7a, all meteorological parameters (with the exception of WS), PM10 mass concentrations, and *Olea*, *Brassica*, *Beta*, and *Panicum* among the Streptophyta genera appear to have significantly contributed to the first two components in the reported ordination plot. 

The T arrow direction is consistent with the location of summer (red) samples, because T reached the highest values in that season. The correlation arrow associated with *Panicum*, which reached the highest RA in summer, suggests that it is strongly correlated with T. A similar result was also found for *Lupinus* and *Physcomitrella*, but their smaller correlation arrows highlight a lower correlation with T. The *Olea* and *Beta* correlation arrows—and, to a lesser extent, the ones associated with *Sesamum*, *Capsicum*, and *Nicotiana*—indicate that they are all intercorrelated, and also correlated with CR and RH. Note that the CR and RH correlation arrows are close to the S3, S12, and S34 samples, in which they reached rather high values. The long correlation arrows associated with PM10, P, and the genus *Brassica* (and, to a lesser extent, *Gossypium*) assume a similar direction in the PCoA plot, highlighting their intercorrelation. More specifically, the arrows of PM10, P, and the genus *Brassica* are close to the S7 and S8 winter samples, likely as a result of the high values of PM10 mass concentration and *Brassica* RA in S7 and S8.

The two-dimensional PCoA plot based on the *BC_i,j_* distances of the nine and three most abundant Ascomycota and Basidiomycota genera’s RAs, respectively, meteorological parameters, and PM10 mass concentrations is shown in Figure 7b. PM10 samples collected in different seasons are on average located in different quadrants of the PCoA plot, contrary to what was found for Streptophyta genera and displayed in Figure 7a. More specifically, the PM10 samples collected in summer and spring are on average located in the lower-right and -left quadrants, respectively, of the PCoA plot (Figure 7b), while the winter and autumn samples are mainly located in the upper-left and -right quadrants, respectively. The greater seasonal impact of the 37 samples on the Ascomycota and Basidiomycota genera structures likely contributed to this result, as Figure 6 also indicates. The length of the correlation arrows in Figure 7b shows that all the meteorological parameters (with the exception of WS) and PM10 mass concentrations appear to have determined the most significant contributions to the first two components of the obtained ordination plot, similarly to what was found for Streptophyta genera (Figure 7a). Nevertheless, most of the Ascomycota (*Botrytis*, *Scheffersomyces*, *Pochonia*, *Sugiyamaella*, and *Colletotrichum*), and the three Basidiomycota (*Ustilago*, *Malassezia*, and *Cryptococcus*) genera, also appear to have significantly contributed. The T arrow direction and length indicate a strong correlation with *Ustilago*, which reached the highest RA values (Figure 6a) in S11 (69.50%) and S18 (35.46%). WS is likely correlated with *Botrytis*, which reached the highest RA in spring (Figure 5b), as its location in the PCoA plot shows. The *Cryptococcus*, *Scheffersomyces*, and *Pochonia* arrow directions and lengths indicate their intercorrelation, as well as that with CR, which reached the highest value in S3 and one of the highest values in S34. The *Malassezia* and, to a lesser extent, the *Fusarium* and *Aspergillus* correlation arrows highlight their intercorrelation, and that with RH. The PCoA plot also highlights the strong correlations of PM10 (and, to a lesser extent, of P) with *Thielavia*, which reached the highest RA in summer (Figure 5b). In addition, the reported PCoA plot shows that *Sugiyamaella* is correlated with both *Colletotrichum*, which reached the highest RA in autumn, and, to a lesser extent, with *Neurospora*.

In conclusion, both of the PCoA plots—and mainly the one concerning Fungi genera (Figure 7b)—proved that the strongest correlations generally occurred between parameters reaching high RAs/values in the same season or PM10 sample, defining an appropriate clustering of the investigated variables.

### 2.6. Relationships among Streptophyta and Ascomycota/Basidiomycota Genera, and with Meteorological Parameters and PM10 Mass Concentrations, by Spearman’s Correlation Coefficients

The 12 most abundant and pervasive Streptophyta genera, and Ascomycota/Basidiomycota genera, were not normally distributed, according to the one-sample Kolmogorov–Smirnov test (Table S8). Consequently, the relationships between plant and fungal genus RAs, and with meteorological parameters and PM10 mass concentrations, were also investigated by Spearman’s correlation coefficients, as reported in Table S9, where values significant at a *p*-levels < 0.05 and 0.01 are in bold and in bold–italic, respectively. The goal of this last analysis was to compare relationships identified by means of significant correlation coefficients with the corresponding ones obtained via the PCoA plots, in addition to highlighting the correlations between Streptophyta and Ascomycota/Basidiomycota genera, and among meteorological parameters. Table 3 summarizes significant positive correlations based on Spearman’s coefficients. Temperature was correlated with *Panicum* (***0.50***) in accordance with Figure 7a, but it did not show any significant correlations with other genera, unlike the one with *Physcomitrella* and *Ustilago* highlighted by the PCoA plots in Figure 7a,b, respectively. The correlation of RH with the Streptophyta genus *Olea* (**0.39**) and the Basidiomycota *Malassezia* (**0.36**) was in accordance with the plots of Figure 7a,b, respectively, but it was in contrast with the correlation of *Pochonia* and *Beta* with RH displayed by Figure 7a,b, respectively. CR and RH were strongly correlated (**0.36**), as shown by the PCoA plots. CR was also correlated with the Streptophyta genera *Olea* (**0.36**) and *Beta* (***0.49***), and the Ascomycota *Pochonia* (***0.43***) and *Scheffersomyces* (***0.60***), in good accordance with the PCoA results. WS was strongly correlated with *Botrytis* (**0.39**), but not with *Ustilago,* as Figure 7b shows. The strong correlation of WS with *Pochonia* (**0.39**) was also contrasting with the outcomes shown in Figure 7b. PM10, in addition to being strongly correlated with P (**0.33**), was also strongly correlated with *Thielavia* (**0.34**), as shown in Figure 7b, and *Gossypium* (**0.33**)—but not with *Brassica*, as Figure 7a displays. 

Few positive correlations occurred among Streptophyta genera. *Olea* was strongly correlated with *Sesamum* (**0.37**)—likely because both genera are naturalized in warm temperate regions of the Middle East, Southern Europe, and Africa—and reached the highest RA in spring (Figure S2). *Beta*, which reached the highest RA in autumn (Figure 5a), was not correlated with *Olea*, in contrast to Figure 7a, but it was correlated with *Nicotiana* (**0.33**), *Cicer* (***0.66***), and *Physcomitrella* (**0.41**), as in Figure 7a. Note that these four genera were all characterized by a similar seasonal trend, since they reached on average the highest and smallest RAs in autumn and spring, respectively. As in Figure 7a*, Sesamum* was also correlated with *Gossypium* (**0.34**), *Capsicum* (***0.45***), and *Nicotiana* (***0.62***). The three genera were all characterized by a similar seasonal dependence. 

Several positive correlations occurred between Streptophyta and Ascomycota/Basidiomycota genera, as shown in Table 3. *Brassica* was correlated with *Aspergillus* (**0.38**) and *Scheffersomyces* (**0.38**), and the three genera all reached the highest RAs in winter. The correlation between *Panicum* and *Ustilago* (***0.43***) could also be due to the similar seasonality: both genera reached the highest RAs in summer and spring. Indeed, all of the correlations between Streptophyta and Ascomycota/Basidiomycota genera listed in Table 3 were likely the result of a rather similar seasonal dependence between the correlated genera. 

Most of the correlations occurring between Ascomycota and Basidiomycota genera also resulted from a similar dependence on seasons. *Colletotrichum* was correlated with *Thielavia* (**0.41**), *Sugiyamaella* (***0.45***), and *Neurospora* (***0.48***), in good accordance with Figure 7b plot, and these were all characterized by similar seasonality (Figure 5b and S6). *Sugiyamaella* and *Malassezia* (**0.38**), which reached the highest RA in autumn (Figure 5b), were also strongly correlated (**0.38**), as Figure 7b shows. In addition, *Thielavia* was also correlated with *Aspergillus* (**0.40**), in contrast to Figure 7b, while *Aspergillus* was correlated with *Pochonia* (**0.35**) and *Neurospora* (***0.44***) because of their similar seasonal dependence. Furthermore, *Pochonia* was correlated with *Scheffersomyces* (***0.47***), *Fusarium* (***0.45***), and *Cryptococcus* (**0.39**), which was also correlated with *Botrytis* (***0.57***), in good accordance with the outcomes displayed by Figure 7b. In conclusion, most of the relationships displayed in Figure 7 were in good accordance with the ones obtained by Spearman’s correlation coefficients. The contrasting correlations between the PCoA plots and the ones listed in Table 3 from Spearman’s correlation coefficients were likely a consequence of the PCoA exploratory analysis, which allows a graphical representation of the similarity/dissimilarity among several different parameters in a single plot. On the other hand, Spearman’s correlation coefficients provide a correlation between two ranks.

### 2.7. Potential Pathogenic Fungi and Plant-Derived Allergens in PM10 Samples

Romano et al. [19] recently provided a preliminary local database on the potential airborne pathogenic bacterial species in PM10 samples collected at the study site. Airborne fungi constitute a substantial fraction of bioaerosols in the atmosphere, as mentioned [31], and there is also a growing attention to the potential harmful effects on living organisms—mostly humans and plants—by fungal bioaerosols themselves. Among the 12 most abundant Fungi genera (mean within-sample RA ≥ 0.95%) detected in the 37 PM10 samples (Figure 6a), we identified 4 potential pathogenic taxa, using the American Biological Safety Association (ABSA) international database [32,33]; the 4 fungal genera in question were *Aspergillus*, *Botrytis*, and *Fusarium*—belonging to the Ascomycota phylum—and *Cryptococcus*, belonging to the Basidiomycota phylum. In the ABSA database, the genus *Aspergillus* is classified at biological safety level 2 (BSL2), and includes pathogenic species for humans, animals, and plants, such as *A. flavus*, *A. fumigatus*, and *A. niger*. Some *Aspergillus* species may cause rot on living plant tissues and/or a variety of allergic reactions and life-threatening systemic infections in humans [34,35,36]. Conflicting reports on seasonal effects on airborne *Aspergillus* spp. levels were reported [37,38]. In our study, the *Aspergillus* genus was detected in all samples, exhibiting the highest and lowest RAs in winter (11.40%) and spring (1.45%), respectively (Figure S6). *Botrytis* is a genus including about 30 different species, well known as fungal phytopathogens, and can be found on a wide variety of agricultural and horticultural plants, thus negatively affecting the production of various crops (vegetables, fruits, field crops, ornamental plants, etc.) [39]. In particular, *Botrytis cinerea* is one of the most common plant pathogens that was detected in all of our PM10 samples. *B. cinerea*, also known as grey mould or *Botrytis* rot, has the ability to thrive in different environments from tropical to cold regions, and infects more than 200 different plant hosts [40], but can also be harmful to humans. Recently, in addition to its allergenic effects [41], Hashimoto et al. [42] reported the first case of pulmonary *Botrytis* sp. infection in an apparently healthy individual. Monteil et al. [43] investigated the role of precipitation (snowfall and rainfall) in the aerial dissemination of *B. cinerea*, and found that its presence is not related to the air mass origin, and is more likely due to below-cloud scavenging. They carried out this field study from December 2005 to November 2011 at 14 sites, mostly in Southern France, and found out that the presence of *B. cinerea* in precipitation was promoted by acidic substances, in addition to the fact that snowfall and rainfall were equal in their deposition capacity, and that high humidity and colder temperatures favoured its contribution. In fact, as we also noticed, the *Botrytis* genus is more abundant in spring, when several days of cloudy and rainy weather, and cool nights, create an ideal environment for *Botrytis* spore germination, infection, and disease development [40,41,44]. Note that the *Botrytis* RA reached higher values in winter and spring (Figure 6a), when T and RH were characterized by lower and higher mean values, respectively, than in summer (Table 1). Blanco et al. [45] also investigated the relationship between concentrations of *B. cinerea* in air and under different environmental conditions, and its incidence in strawberry flowers and fruits in Huelva (Spain). They observed that the *B. cinerea* concentration was significantly and positively correlated with the average solar radiation and mean temperature, and negatively with rainfall and relative humidity, in contrast to the findings of Monteil et al. [43] and of this study. *Fusarium*, which comprises widespread filamentous fungi [46], was the least abundant potential pathogenic genus (Figure 5b) in every season; it reached the highest RA in winter, with a mean value of 1.61%, and the lowest RA in spring, with a mean value of 0.36% (Figure S6). *Fusarium* species are primarily plant pathogens, but they can also infect a human host, inducing local and, rarely, systemic infections, especially in immunocompromised patients [47]. The genus *Cryptococcus* includes at least 37 different species, of which two—*C. neoformans* and *C. gattii*—are recognized as important human and animal pathogens, causing highly infectious respiratory mycosis [48,49]. In our samples, *Cryptococcus* appeared evenly distributed throughout the year, except for the summer, when its RA was about 10 times lower than in the other seasons (Figure S6). In addition to the potentially pathogenic fungal genera listed in the ABSA database, we identified other Fungi genera that are reportedly potential pathogens, according to previous studies, e.g., the Ascomycota *Candida* [13,24] and *Colletotrichum* [50], and the Basidiomycota *Ustilago* [51]. The genus *Colletotrichum* is ranked as the eighth most devastating plant-pathogenic fungus in the world; it consists of many species causing plant disease on a wide range of plants, covering both woody and herbaceous plants. The occurrence and effects of anthracnose disease caused by *Colletotrichum* species are very common in tropical and subtropical areas, where the climatic conditions are warm and humid, but recent research has shown some high-profile species of *Colletotrichum* surviving in temperate regions and, thus affecting temperate crops [52]. Valle-Aguirre et al. [53] investigated the presence of fungal colonies in an agroecosystem of avocado trees in Mexico. Thirty-two airborne fungal genera were identified and, among them, *Fusarium* (97.2%) and *Colletotrichum* (94.4%) were the most common fungal pathogens in the avocado orchard atmosphere, with their contributions being highest in June. We found in our study that *Colletotrichum* reached the highest RA in summer (29.04%), and that, in addition to the two aforementioned genera, *Aspergillus* was the other fungal pathogen genus in common with those identified by Valle-Aguirre et al. [53]. The *Ustilago* genus is represented by more than 400 cosmopolitan species, which are parasitic and infect the floral parts of wheat, barley, oat, maize, sugarcane, and wild grasses. The geographical distribution of the *Ustilago* disease involves temperate areas of the world such as North India, Siberia, Europe, and North and South America [51].

Aside from potentially pathogenic Fungi, air may also transport several plant-derived allergens that can be responsible for respiratory diseases. Aeroallergens are carried by plant-derived particles, such as pollen grains or paucimicronic plant-derived components, acting as carriers for the protein agent with antigenic properties that cause symptoms in predisposed subjects [54]. We used the AllerBase Allergen Database [55,56] to screen for allergen-producing plants in our PM10 samples. With the exceptions of *Panicum* and *Physcomitrella*, all of the most abundant and pervasive Streptophyta genera reported in Figure 4a were included in the AllerBase database, indicating a massive and constant presence of aeroallergens circulating in the studied area.

## 3. Summary and Conclusions

The high-throughput sequencing of the 18S rRNA gene was applied to the DNA extracted from 37 airborne PM10 samples, collected from July 2018 to June 2019 at a coastal site in Southern Italy, with the main goal of providing a preliminary dataset on the seasonal and meteorological parameter dependence of the airborne eukaryotic taxonomic biodiversity. 

Viridiplantae and Fungi were the most abundant eukaryotic kingdoms. Viridiplantae were prevailing and reached the highest contribution (72.26%) in winter, while Fungi reached the highest contribution (23.91%) in autumn. Streptophyta was the prevailing Viridiplantae phylum, and Ascomycota and Basidiomycota were the prevailing Fungi phyla. Ascomycota reached the highest percentage contribution (82.62%) in summer, while Basidiomycota in winter (35.63%).

The total number of OTUs and genera were weakly affected by seasons. In contrast, the Shannon and Simpson indices for Viridiplantae and Fungi at the genus level were characterized by a strong seasonal dependence, because of the strong day-by-day and seasonal dependence of the genus community structure. 

The relative abundance of the 12 most abundant and pervasive Streptophyta and Ascomycota/Basidiomycota genera was analysed to characterize the genus community in the 37 analysed PM10 samples. The 12 most abundant and pervasive Streptophyta genera varied day-by-day and with seasons in the 37 samples. *Brassica* and *Panicum* were the most abundant genera in winter (43.13%) and summer (27.35%), respectively. *Olea* was the most abundant genus in spring (32.71%) and autumn (20.82%). Nine Ascomycota and three Basidiomycota genera made up the twelve most abundant and pervasive Fungi genera. *Botrytis* and *Colletotrichum* were the predominant Ascomycota genera, reaching the highest RA in spring (53.35%) and summer (29.04%), respectively, whereas *Cryptococcus* and *Ustilago* were the prevailing Basidiomycota genera, with the highest RA equal to 18.52% and 17.89% in winter and spring, respectively.

PCoA plots and non-parametric Spearman’s rank-order correlation coefficients were used to characterize the relationships between genera, and with meteorological parameters and PM10 mass concentrations. Few strong positive relationships between genera and meteorological parameters were found, according to Spearman’s correlation coefficients, in contrast to the strong seasonal dependence of the genus RAs. *Panicum*, which reached the highest RA in summer, was the only Streptophyta significantly correlated with T. Similarly, the Basidiomycota *Ustilago*, which reached the highest RA in spring, was the only Fungi genus correlated with T. *Olea* and *Beta* were the only Streptophyta genera significantly correlated with CR. The Ascomycota *Pochonia* and *Scheffersomyces* were the only Fungi genera correlated with CR. Moreover, *Pochonia* and *Botrytis* were the only Fungi genera correlated with WS. The Basidiomycota *Malassezia* was the only genus strongly correlated with RH; it reached the highest RA in autumn, when RH also reached the highest mean value. Finally, the Streptophyta *Gossypium* and the Ascomycota *Thielavia* were the only genera correlated with PM10 mass concentrations. Both genera reached high RAs in PM10 samples characterized by mass concentrations far above the mean value. 

The relationships between Viridiplantae genera or Fungi genera, as well as the relationships between Streptophyta and Ascomycota/Basidiomycota genera, generally occurred between genera characterized by similar seasonality.

Most of the strong and very strong correlations based on Spearman’s correlation coefficients, which provide the correlation between two ranks, were in good accordance with the ones displayed by the PCoA plots. However, PCoA plots displayed a greater number of correlations between different parameters, some of which contrast with those provided by Spearman’ correlation coefficients. This last result may be due to the use of a single framework in PCoA plots to represent similarity/dissimilarity between several different parameters. 

The analysis of samples with an atypical community structure, due to the prevailing RA of a single genus within the samples, allowed us to infer the potential impact of long-range transported air masses on the sample community structure. In fact, the impacts of air masses advected from northern African deserts and/or from the anthropogenically polluted areas in Northern and Eastern Europe was likely detected in some samples. 

Finally, special attention was also given to potentially pathogenic fungus- and plant-derived allergens, because of their potential harmful effects on living organisms—mostly humans and plants. Seven potential pathogenic genera of Fungi (*Aspergillus*, *Botrytis*, *Candida*, *Colletotrichum, Cryptococcus, Fusarium*, and *Ustilago*) were detected in our PM10 samples. Then, the screening of the allergen-producing plants showed that, with the exception of *Panicum* and *Physcomitrella*, all of the most abundant and pervasive Streptophyta genera detected were responsible for the presence of aeroallergens in the studied area.

In conclusion, a preliminary dataset on the airborne eukaryotic community structure and its dependence on seasons and meteorological parameters at a coastal site of southeast Italy was provided. To the best of our knowledge, there are no previous studies on the eukaryotic community structure related to this area, which can be considered to be representative of coastal sites of the Central Mediterranean. Therefore, our findings may be of great interest, since they can contribute to the research on the dissemination of human, animal, and plant diseases and pandemics, and to the design of efficient regulations and policies for environmental protection and public health in the quite important Central Mediterranean region.

## 4. Material and Methods

### 4.1. Sampling Site, PM10 Sample Collection, and Meteorological Data

PM10 samples were collected at about 10 m above ground level, on the roof of the Mathematics and Physics Department of the University of Salento, which is located in a suburban site (40.3°N; 18.1°E) in Lecce (southeast Italy), in the flat Salento Peninsula (Figure S1). A low-volume (2.3 m^3^ h^−1^) HYDRA-FAI dual channel sampler was used to collect PM10 particles on 47-mm-diameter PTFE (polytetrafluoroethylene) filters (TEFLO W/RING 2 μ from VWR International S.R.L.), which showed excellent collection efficiency [57]. Forty-three PM10 samples were collected from July 2018 to June 2019 by performing 24- or 48-h samplings. We tested different sampling times to investigate the sensitivity of the 18S rRNA metabarcoding analysis to the detection of the airborne eukaryotic community collected in the sampled PM10 mass. We also assumed that the eukaryotic community’s growth or decay was negligible during the sampling period. After sampling, each filter was put in a sterile box and stored at −20 °C, since eukaryotic community growth is assumed to be unlikely at such temperature [58]. Three control filters—which were not subjected to sampling, but handled and stored in the same way as the sampled filters—were used as negative controls. 

Meteorological parameters were monitored a few hundred meters away from the study site, and were provided by Istituto di Scienze dell’Atmosfera e del Clima—ISAC-CNR (Lecce, Italy) (http://www.basesperimentale.le.isac.cnr.it/, accessed on 15 December 2020). More specifically, measurements from the aforementioned meteorological station, co-located in time with the PM10 samplings, were used to calculate 24- or 48-h mean values of temperature (T), relative humidity (RH), atmospheric pressure (P), wind direction and speed (WD and WS, respectively), and cumulative rain (CR) during the sampling time.

### 4.2. Long-Range Transported Air Masses at the Study Site

The study site is located on a narrow peninsula in the Central Mediterranean basin (Figure S1) and, as several studies showed [18,20,22,59,60,61,62], it is affected by long-range transported aerosols. More specifically, it is affected by desert dust from northern Africa, polluted particles from urban and industrial areas of Northern and Eastern Europe, marine aerosols from the Mediterranean Sea and the Atlantic Ocean, and biomass-burning particles from forest fires occurring mainly in summertime across Central Mediterranean sites. A detailed analysis of the main airflows at the study site by means of the 4-day back trajectories from the Hybrid Single-Particle Lagrangian Integrated Trajectory (HYSPLIT) model, version 4.8, from NOAA/ARL (https://www.ready.noaa.gov/, accessed on 14 December 2020) [63], was provided by [17,60]. Data from the BSC_DREAM8b model (https://www.bsc.es/, accessed on 11 December 2020) [64] were also used to support the advection of desert dust particles at the study site, where Romano et al. [18] have recently analysed the impact of the long-range transported air masses on the bacterial community structure.

### 4.3. DNA Extraction and 18SrRNA Gene High-Throughput Sequencing

Plant DNA recovered from PM filters likely derived from pollen grains and plant debris. Fungal DNA detected in aerosol filter samples derived mostly from spores—which are known to have very small size, resist environmental stress, and be potentially transported over longer distances—but it can also originate from other fungal material, such as hyphae and tissue fragments [65]. Airborne eukaryotes were recovered in this study from PM10 PTFE filters in aseptic conditions, as described in detail by Romano et al. [18]. More specifically, each filter, once cut into 10–15 strips, was placed in a 50-mL conical Falcon tube containing a 40-mL solution made up of PBT (0.003% Tween-20, 17 mmol L^−1^ KH_2_PO_4_, and 72 mmol L^−1^ K_2_HPO_4_). The Falcon tube with the filter strips in solution was vortexed for 5 min at maximum power and sonicated at room temperature. Then, the suspension was poured into a clean Falcon tube. The wash was repeated with an additional 40 mL of PBT in order to remove any residual material from the filter. Both sample washes were centrifuged for 30 min at 3500× *g* to recover eukaryotes. The pellets were processed for DNA extraction using the DNeasy PowerSoil kit (Qiagen, Milan, Italy). Eluted DNA was precipitated in 10 mM of TrisHCl, pH8.

Next-generation sequencing (NGS) experiments, comprising sample quality control and bioinformatics analyses, were performed by Genomix4life S.R.L. (Baronissi, Salerno, Italy). The final yield and quality of extracted DNA were determined using a NanoDrop ND-1000 spectrophotometer (Thermo Scientific, Waltham, MA, USA) and a Qubit Fluorometer 1.0 (Invitrogen Co., Carlsbad, CA). Then, 18S amplification was performed with primers: NS1: 5’-GTAGTCATATGCTTGTCTC-3’, and NS2: 5’-GGCTGCTGGCACCAGACTTGC-3’. The NS1 and NS2 primers were designed to amplify a region of approximately 515 bp within 18S rDNA from many fungi, protozoans, algae, plants, and animals (the size of the amplified region plus the primers is approximately 555 bp) [66]. No amplification product was observed in the negative control. Each PCR reaction was assembled according to the Metagenomic Sequencing Library Preparation (Illumina, San Diego, CA, USA) protocol. Libraries were quantified using a Qubit fluorometer (Invitrogen Co., Carlsbad, CA, USA) and pooled to an equimolar amount of each index-tagged sample to a final concentration of 4 nM, including the PhiX Control Library (Illumina; expected 30%). Pooled samples were subjected to cluster generation and sequenced on the MiSeq platform (Illumina, San Diego, CA, USA) in a 2 × 250 paired-end format. The raw sequence files generated (fast files) underwent quality control analysis via FastQC. The 18S metagenomics analysis was performed with Kraken, which assigns taxonomic labels to short DNA sequences with high sensitivity and speed, using exact alignments of k-mers and a novel classification algorithm [67]. The database for eukaryotes was composed of RefSeq-complete genomes/proteins [68].

Among the 43 PM10 samples analysed through 18S rRNA gene metabarcoding, the sequencing failed in 6 of the 24-h PM10 samples due to the low amount of the extracted DNA.

### 4.4. Statistical Analyses and Software

Statistical analyses were performed using the data from all 37 samples, in order to characterize and compare eukaryotic communities and investigate the relationships between them and with meteorological parameters and PM10 mass concentrations. The biodiversity of the analysed 37 samples was evaluated using the Shannon *H* [69] and Simpson *D* [70] indices. They represent the most common parameters used to quantify and describe the population (alpha) diversity in different types of samples [18,71,72,73,74]. The Shannon index is mostly related to species richness (total number of identified species), giving more importance to rare species [75]. On the other hand, the Simpson index is associated with species evenness (in turn associated with species relative abundance) more than species richness, giving a greater weight to species with more frequency in a sample [73,76]. In more detail, the Shannon *H* and the Simpson *D* indices are calculated as follows:

*H* = *Σ_i_ p_i_* ln *p_i_* (1)

*D* = *Σ_i_* (*p_i_*)^2^ (2)

where *p_i_* can be expressed as *n_i_ /N* for a well-sampled community, with *n_i_* representing the number of individuals of the species *i*, and *N* the corresponding total number in the community [75]. Therefore, *H* increases as the richness of the community also increases, whereas *D* represents a value between 0 and 1, indicating a non-diverse community if *D* = 1, and an infinitely diverse community if *D* = 0 [77]. We also evaluated the dissimilarity between all of the possible pairings of samples in our dataset using the Bray–Curtis dissimilarity indices (*BC*), based on the relative abundances of the eukaryotic community components. *BC* indices represent the most common measure of beta-diversity, and can be generally estimated by the following expression:

*BC_i,j_* = |*S_i_* − *S_j_*|/(*S_i_* + *S_j_*) (3)

with *i* and *j* indicating the two investigated samples, whereas *S_i_* and *S_j_* represent the total number of species identified in samples *i* and *j*, respectively [78]. The *BC_i,j_* dissimilarity index assumes a value between 0 and 1: the two investigated samples share all of the same species if *BC _i,j_* = 0, whereas they do not share any species if *BC _i,j_* = 1.

The one-sample Kolmogorov–Smirnov test (using the MATLAB *kstest* function) was used to test whether the investigated parameters were abnormally distributed. Then, the relationships between eukaryotic community components, PM10 mass concentrations, and meteorological parameters were analysed by the non-parametric Spearman’s rank-order correlation coefficients, if the data were not normally distributed. The PAST (Paleontological Statistics) software package (Version 4.03) [79] was used to calculate both *BC_i,j_* matrices and Spearman’s correlation coefficients.

The principal coordinates analysis (PCoA) technique was also used to analyse the relationships among the 37 investigated samples. The PCoA represents one of the most popular exploratory analyses, allowing the graphical representation of the similarity (or dissimilarity) among values of multiple variables [30] The ordination method aims at representing the variables in a new system of coordinates trying to summarize the original information of the data (i.e., the variance) in a reduced space (score plot). More specifically, PCoA components represent a complex function of original variables, depending on the selected measure of dissimilarity, expressed as a distance matrix [80]. The PCoA performance can be evaluated using the percentage of total variance explained by the first two or three axes related to the corresponding components. In addition to the score plot, another output of the PCoA technique is the correlation circle plot, in which longer arrows represent variables that contribute significantly to either one or two PCoA components. Stronger correlations between two variables are associated with a greater absolute value of the cosine of the angle between the two corresponding arrows and, therefore, arrows in the same and in the opposite direction suggest a positive or a negative correlation, respectively [80]. The *BC_i,j_* matrix was used in our study as input parameter for the PCoA analysis. In fact, note that non-Euclidean distances, such as the *BC* distance, can also be used as inputs for PCoA, but with the inclusion of an offset to remedy possible negative percentages of variance explained (i.e., negative eigenvalues) [80]. More specifically, the PCoA ordination method using *f_pcoa* and *f_pcoaPlot* functions with an offset correction [81] was applied to the selected datasets using the MATLAB Fathom Toolbox [82].

## Figures and Tables

**Table 1:** Seasonal mean values (± standard deviation) of the PM10 mass concentration and meteorological parameters in winter (January, February, and March: samples S1–S8), spring (April, May, and June: samples S9–S15), summer (July, August, and September: samples S16–S20), and autumn (October, November, and December: samples S21–S37). T, RH, P, WD, and WS show the seasonal mean values of air temperature, relative humidity, atmospheric pressure, wind direction, and wind speed, respectively. CR provides the cumulative rain.

Season	PM10	T	RH	P	CR	WD	WS
	(µg m^−3^)	(°C)	(%)	(mbar)	(mm)	(deg)	(ms^−1^)
Winter (mean ± SD)	25 ± 15	8.7 ± 1.6	65 ± 13	1012.4 ± 11.5	39.1	341 ± 31	2.4 ± 1.4
Spring (mean ± SD)	20 ± 5	16.9 ± 3.4	72 ± 7	1011.4 ± 4.2	29.2	126 ± 59	2.2 ± 0.9
Summer (mean ± SD)	24 ± 4	26.1 ± 1.1	57 ± 5	1009.5 ± 3.3	0.0	348 ± 34	2.0 ± 0.9
Autumn (mean ± SD)	22 ± 11	12.7 ± 4.6	76 ± 9	1013.9 ± 4.6	32.6	329 ± 8	1.5 ± 1.0

**Table 2:** Number (n^o^) of Viridiplantae and Fungi operational taxonomic units (OTUs) and genera for the 37 analysed samples. Shannon (*H*) and Simpson (*D*) indices at the genus level are also reported for both kingdoms.

Sample	Viridiplantae				Fungi			
	n° OTUs	n° Genera	At Genus Level		n° OTUs	n° Genera	At Genus Level	
			Shannon Index (*H*)	Simpson Index (*D*)			Shannon Index (*H*)	Simpson Index (*D*)
S1	143	42	2.33	0.14	77	19	2.26	0.13
S2	149	43	2.33	0.14	66	14	1.90	0.18
S3	142	42	1.98	0.21	77	18	2.02	0.18
S4	134	39	2.04	0.19	67	16	2.00	0.21
S5	141	41	1.94	0.23	76	18	2.02	0.21
S6	140	39	2.28	0.20	70	16	2.11	0.17
S7	142	40	0.91	0.69	58	13	2.02	0.17
S8	142	42	1.04	0.65	60	14	2.18	0.14
S9	150	44	2.08	0.20	65	15	1.81	0.24
S10	140	40	2.34	0.15	73	17	1.42	0.37
S11	144	42	2.04	0.22	75	18	1.06	0.52
S12	140	41	2.05	0.21	76	18	1.41	0.42
S13	145	43	2.13	0.18	73	17	1.10	0.57
S14	140	41	1.77	0.29	67	16	1.61	0.31
S15	145	43	1.80	0.26	76	18	1.08	0.55
S16	144	43	1.71	0.35	72	17	1.67	0.28
S17	140	41	2.43	0.12	69	16	1.74	0.24
S18	152	45	2.17	0.18	76	18	1.95	0.20
S19	139	42	2.01	0.25	68	16	1.73	0.23
S20	150	44	2.25	0.15	71	16	1.80	0.23
S21	147	44	2.09	0.17	77	18	2.27	0.12
S22	137	40	2.07	0.20	76	18	1.68	0.26
S23	130	37	2.29	0.14	73	17	1.30	0.43
S24	150	44	2.26	0.15	72	16	1.83	0.23
S25	143	42	2.10	0.17	75	18	1.93	0.20
S26	128	37	1.97	0.22	69	16	1.44	0.40
S27	140	41	2.13	0.17	73	17	2.33	0.11
S28	106	30	1.41	0.44	61	14	1.52	0.27
S29	142	42	2.25	0.15	74	17	2.33	0.12
S30	128	37	1.83	0.24	67	17	1.46	0.37
S31	146	43	2.08	0.17	76	18	2.18	0.15
S32	146	43	1.89	0.25	69	16	2.07	0.18
S33	131	39	2.43	0.13	65	15	1.98	0.18
S34	130	37	1.87	0.25	66	14	2.15	0.16
S35	146	43	2.41	0.12	75	18	2.30	0.12
S36	143	43	2.31	0.14	66	16	2.01	0.20
S37	146	43	1.95	0.23	74	18	1.83	0.22

**Table 3:** Relationships between the 12 most abundant and pervasive Streptophyta (green) and Ascomycota/Basidiomycota (red) genera, meteorological parameters (blue), and PM mass concentrations (grey) in the analysed samples. The related Spearman’s correlation coefficient is reported in brackets (values significant at a *p*-level < 0.05 and 0.01 are in bold and bold–italic, respectively).

Streptophyta Genera	Positive Correlations	Fungi Phyla	Fungi Genera	Positive Correlations
*Brassica* *(BRA)*	***ASP*** (**0.38**), ***SCHE*** (**0.38**), **P** (**0.33**)	ASCOMYCOTA	*Botrytis* *(BOT)*	***CRY*** (***0.57***), **WS** (**0.39**)
*Olea* *(OLE)*	***SES*** (**0.37**), **RH** (**0.39**), **CR** (**0.36**)		*Colletotrichum* *(COL)*	***THI*** (**0.41**), ***SUG*** (***0.45***), ***NEU*** (***0.48***)
*Panicum* *(PAN)*	***UST*** (***0.43***), **T** (***0.50***)		*Thielavia* *(THI)*	***COL*** (**0.41**), ***SUG*** (***0.47***), ***ASP*** (**0.40**), **PM10** (**0.34**)
*Beta* *(BET)*	***PHY*** (**0.41**), ***NIC*** (**0.33**), ***CIC*** (***0.66***), ***COL*** (**0.36**), ***NEU*** (**0.34**), ***SCHE*** (***0.53***), ***MAL*** (**0.40**), **CR** (***0.49***)		*Sugiyamaella* *(SUG)*	***THI*** (***0.47***), ***MAL*** (**0.38**)
*Physcomitrella* *(PHY)*	***BET*** (**0.41**), ***CIC*** (**0.40**), ***COL*** (***0.60***), ***THI*** (***0.44***), ***SUG*** (***0.46***), ***ASP*** (**0.37**), ***NEU*** (***0.60***)		*Aspergillus* *(ASP)*	***THI*** (**0.40**), ***POC*** (**0.35**), ***NEU*** (***0.44***), ***FUS*** (***0.73***)
*Gossypium* *(GOS)*	***SES*** (**0.34**), **PM10** (**0.33**)		*Pochonia* *(POC)*	***ASP*** (**0.35**), ***SCHE*** (***0.47***), ***FUS*** (***0.45***), ***CRY*** (**0.39**), **CR** (***0.43***), **WS** (**0.39**)
*Capsicum* *(CAP)*	***SES*** (***0.45***), ***BOT*** (***0.51***), ***POC*** (**0.41**)		*Neurospora* *(NEU)*	***COL*** (***0.48***), ***ASP*** (***0.44***), ***FUS*** (***0.43***)
*Nicotiana* *(NIC)*	***BET*** (**0.33**), ***SES*** (***0.62***), ***CIC*** (***0.48***), ***MAL*** (**0.36**)		*Scheffersomyces* *(SCHE)*	***POC*** (***0.47***), ***FUS*** (**0.39**), ***CRY*** (***0.48***), **CR** (***0.60***)
*Daucus* *(DAU)*	***NEU*** (**0.35**)		*Fusarium* *(FUS)*	***ASP*** (***0.73***), ***POC*** (***0.45***), ***NEU*** (***0.43***), ***SCHE*** (**0.39**)
*Sesamum* *(SES)*	***OLE*** (**0.37**), ***GOS*** (**0.34**), ***CAP*** (***0.45***), ***NIC*** (***0.62***), ***BOT*** (**0.36**)	BASIDIOMYCOTA	*Cryptococcus* *(CRY)*	***BOT*** (***0.57***), ***POC*** (**0.39**), ***SCHE*** (***0.48***)
*Cicer* *(CIC)*	***BET*** (***0.66***), ***PHY*** (**0.40**), ***NIC*** (***0.48***), ***SUG*** (**0.35**)		*Ustilago* *(UST)*	**T** (**0.37**)
*Lupinus* *(LUP)*	***ASP*** (**0.38**), ***UST*** (***0.47***)		*Malassezia* *(MAL)*	***SUG*** (**0.38**), **RH** (**0.36**)
T			CR	**RH** (**0.36**)
RH	**CR** (**0.36**)		WS	
			PM10	**P** (**0.33**)

## Data Availability

Not applicable.

## Supplementary Materials

The following are available online at https://www.mdpi.com/article/10.3390/toxins13080518/s1, Figure S1: Geographical location of the monitoring site; Figure S2: Mean contribution of the Streptophyta genera; Figure S3: Analytical back trajectories on 21 and 28 February 2019; Figure S4: Analytical back trajectories on 21 and 22 November 2018; Figure S5: Dust load map from the BSC-DREAM8b model on 21 November 2018; Figure S6: Mean contribution of the Ascomycota/Basidiomycota genera; Figure S7: Analytical back trajectories on 9 May 2019; Figure S8: Dust load map from the BSC-DREAM8b model on 9 May 2019; Figure S9: Analytical back trajectories on 17 and 18 October 2018; Table S1: Summary table of sampling times, PM10 concentration, and meteorological parameters; Table S2: Kolmogorov–Smirnov test statistics for PM10 and meteorological parameter data; Table S3: Heat map of the within-sample relative abundances for the Streptophyta genera; Table S4: Heat map of the within-sample relative abundances for the Fungi genera; Table S5: Seasonal mean values of the number of Viridiplantae and Fungi OTUs and genera, and biodiversity indices; Table S6: Bray–Curtis dissimilarity matrix based on the relative abundances of the Streptophyta genera; Table S7: Bray–Curtis dissimilarity matrix based on the relative abundances of the Ascomycota/Basidiomycota genera; Table S8: Kolmogorov–Smirnov test statistics for the Streptophyta and Ascomycota/Basidiomycota genera; Table S9: Spearman’s correlation coefficients between the Streptophyta and Ascomycota/Basidiomycota genus abundances.

## References

[1] Gusareva E.S., Acerbi E., Lau K.J.X., Luhung I., Premkrishnan B.N.V., Kolundžija S., Purbojati R.W., Wong A., Houghton J.N.I., Miller D. (2019). Microbial communities in the tropical air ecosystem follow a precise diel cycle. Proc. Natl. Acad. Sci. USA.

[2] Womack A.M., Bohannan B.J.M., Green J.L. (2010). Biodiversity and biogeography of the atmosphere. Philos. Trans. R. Soc. B. Biol. Sci..

[3] Núñez A., García A.M., Moreno D.A., Guantes R. (2021). Seasonal changes dominate long-term variability of the urban air microbiome across space and time. Environ. Int..

[4] Romano S., Becagli S., Lucarelli F., Rispoli G., Perrone M. (2020). Airborne bacteria structure and chemical composition relationships in winter and spring PM10 samples over southeastern Italy. Sci. Total Environ..

[5] Maki T., Lee K.C., Kawai K., Onishi K., Hong C.S., Kurosaki Y., Shinoda M., Kai K., Iwasaka Y., Archer S.D.J. (2019). Aeolian Dispersal of Bacteria Associated With Desert Dust and Anthropogenic Particles Over Continental and Oceanic Surfaces. J. Geophys. Res. Atmos..

[6] Falkowski P.G., Fenchel T., Delong E.F. (2008). The Microbial Engines That Drive Earth’s Biogeochemical Cycles. Science.

[7] Gonzalez-Martin C., Schmidt T.M. (2019). Airborne Infectious Microorganisms. Encyclopedia of Microbiology.

[8] Jones R.M., Brosseau L.M. (2015). Aerosol Transmission of Infectious Disease. J. Occup. Environ. Med..

[9] Bourdrel T., Annesi-Maesano I., Alahmad B., Maesano C.N., Bind M.-A. (2021). The impact of outdoor air pollution on COVID-19: A review of evidence from in vitro, animal, and human studies. Eur. Respir. Rev..

[10] Chen X., Kumari D., Achal V. (2020). A Review on Airborne Microbes: The Characteristics of Sources, Pathogenicity and Geography. Atmosphere.

[11] Bowers R.M., Clements N., Emerson J., Wiedinmyer C., Hannigan M., Fierer N. (2013). Seasonal Variability in Bacterial and Fungal Diversity of the Near-Surface Atmosphere. Environ. Sci. Technol..

[12] Gandolfi I., Bertolini V., Ambrosini R., Bestetti G., Franzetti A. (2013). Unravelling the bacterial diversity in the atmosphere. Appl. Microbiol. Biotechnol..

[13] Aziz A.A., Lee K., Park B., Park H., Park K., Choi I.-G., Chang I.S. (2018). Comparative study of the airborne microbial communities and their functional composition in fine particulate matter (PM2.5) under non-extreme and extreme PM2.5 conditions. Atmos. Environ..

[14] Song H., Crawford I., Lloyd J., Robinson C., Boothman C., Bower K., Gallagher M., Allen G., Topping D. (2020). Airborne Bacterial and Eukaryotic Community Structure across the United Kingdom Revealed by High-Throughput Sequencing. Atmosphere.

[15] Ruiz-Gil T., Acuña J.J., Fujiyoshi S., Tanaka D., Noda J., Maruyama F., Jorquera M.A. (2020). Airborne bacterial communities of outdoor environments and their associated influencing factors. Environ. Int..

[16] Calderón-Ezquerro M.D.C., Serrano-Silva N., Brunner-Mendoza C. (2021). Aerobiological study of bacterial and fungal community composition in the atmosphere of Mexico City throughout an annual cycle. Environ. Pollut..

[17] Perrone M., Becagli S., Orza J.A.G., Vecchi R., Dinoi A., Udisti R., Cabello M. (2013). The impact of long-range-transport on PM1 and PM2.5 at a Central Mediterranean site. Atmos. Environ..

[18] Romano S., Di Salvo M., Rispoli G., Alifano P., Perrone M.R., Talà A. (2019). Airborne bacteria in the Central Mediterranean: Structure and role of meteorology and air mass transport. Sci. Total Environ..

[19] Romano S., Fragola M., Alifano P., Perrone M., Talà A. (2021). Potential Human and Plant Pathogenic Species in Airborne PM10 Samples and Relationships with Chemical Components and Meteorological Parameters. Atmosphere.

[20] Pietrogrande M.C., Perrone M.R., Manarini F., Romano S., Udisti R., Becagli S. (2018). PM10 oxidative potential at a Central Mediterranean Site: Association with chemical composition and meteorological parameters. Atmos. Environ..

[21] Perrone M., Romano S., Genga A., Paladini F. (2018). Integration of optical and chemical parameters to improve the particulate matter characterization. Atmos. Res..

[22] Perrone M.R., Romano S., Orza J. (2014). Particle optical properties at a Central Mediterranean site: Impact of advection routes and local meteorology. Atmos. Res..

[23] O’Neill M.A., Darvill A.G., Etzler M.E., Mohnen D., Perez S., Varki A., Cummings R.D., Esko J.D. (2017). Viridiplantae and Algae. Essentials of Glycobiology [Internet].

[24] Banchi E., Ametrano C.G., Tordoni E., Stanković D., Ongaro S., Tretiach M., Pallavicini A., Muggia L., Verardo P., Tassan F. (2020). Environmental DNA assessment of airborne plant and fungal seasonal diversity. Sci. Total Environ..

[25] Núñez A., de Paz G.A., Rastrojo A., Ferencova Z., Gutiérrez-Bustillo A.M., Alcamí A., Moreno D.A., Guantes R. (2019). Temporal patterns of variability for prokaryotic and eukaryotic diversity in the urban air of Madrid (Spain). Atmos. Environ..

[26] Han B., Weiss L.M. (2017). Microsporidia: Obligate Intracellular Pathogens Within the Fungal Kingdom. The Fungal Kingdom.

[27] Du P., Du R., Ren W., Lu Z., Zhang Y., Fu P. (2018). Variations of bacteria and fungi in PM2.5 in Beijing, China. Atmos. Environ..

[28] Banchi E., Pallavicini A., Muggia L. (2019). Relevance of plant and fungal DNA metabarcoding in aerobiology. Aerobiologia.

[29] Shi C.-F., Zhang K.-H., Chai C.-Y., Yan Z.-L., Hui F.-L. (2021). Diversity of the genus Sugiyamaella and description of two new species from rotting wood in China. MycoKeys.

[30] Ramette A.N. (2007). Multivariate analyses in microbial ecology. FEMS Microbiol. Ecol..

[31] Janssen R.H.H., Heald C.L., Steiner A.L., Perring A.E., Huffman J.A., Robinson E.S., Twohy C.H., Ziemba L.D. (2021). Drivers of the fungal spore bioaerosol budget: Observational analysis and global modeling. Atmos. Chem. Phys. Discuss..

[32] Brown E., McTaggart L.R., Low D.E., Richardson S.E. (2014). Effective method for the heat inactivation of Blastomyces dermatitidis. Med. Mycol..

[33] Singh N.K., Wood J.M., Karouia F., Venkateswaran K. (2018). Succession and persistence of microbial communities and antimicrobial resistance genes associated with International Space Station environmental surfaces. Microbiome.

[34] Pfliegler W.P., Pócsi I., Győri Z., Pusztahelyi T. (2020). The Aspergilli and Their Mycotoxins: Metabolic Interactions With Plants and the Soil Biota. Front. Microbiol..

[35] Foley K., Fazio G., Jensen A.B., Hughes W.O. (2014). The distribution of Aspergillus spp. opportunistic parasites in hives and their pathogenicity to honey bees. Vet. Microbiol..

[36] Paulussen C., Hallsworth J.E., Álvarez-Pérez S., Nierman W.C., Hamill P.G., Blain D., Rediers H., Lievens B. (2016). Ecology of aspergillosis: Insights into the pathogenic potency of *Aspergillus fumigatus* and some other Aspergillus species. Microb. Biotechnol..

[37] Guinea J., Peláez T., Alcalá L., Bouza E. (2006). Outdoor environmental levels of *Aspergillus* spp. conidia over a wide geographical area. Med. Mycol..

[38] R H.P., Singh R.K., Akila M., Ravikrishna R., Verma R., Gunthe S.S. (2017). Seasonal variation of the dominant allergenic fungal aerosols–One year study from southern Indian region. Sci. Rep..

[39] Aydoğdu M., Kurbetli I., Canpolat S. (2020). Batı Akdeniz Bölgesi’nde Enginar baş çürüklüğü (*Botrytis cinerea* Pers.) hastalığının yaygınlığı ve üretime etkisinin belirlenmesi. Bitki Koruma Bülteni.

[40] Williamson B., Tudzynski B., Tudzynski P., van Kan J. (2007). Botrytis cinerea: The cause of grey mould disease. Mol. Plant Pathol..

[41] Jurgensen C.W., Madsen A.M. (2009). Exposure to the airborne mould Botrytis and its health effects. Ann. Agric. Environ. Med..

[42] Hashimoto S., Tanaka E., Ueyama M., Terada S., Inao T., Kaji Y., Yasuda T., Hajiro T., Nakagawa T., Noma S. (2019). A case report of pulmonary Botrytis sp. infection in an apparently healthy individual. BMC Infect. Dis..

[43] Monteil C., Bardin M., E Morris C. (2014). Features of air masses associated with the deposition of *Pseudomonas syringae* and *Botrytis cinerea* by rain and snowfall. ISME J..

[44] Elad Y., Williamson B., Tudzynski P., Delen N. (2007). Botrytis spp. and Diseases They Cause in Agricultural Systems–An Introduction. Botrytis: Biology, Pathology and Control.

[45] Blanco C., De Santos B.L., Romero F. (2006). Relationship between Concentrations of Botrytis Cinerea Conidia in Air, Environmental Conditions, and the Incidence of Grey Mould in Strawberry Flowers and Fruits. Eur. J. Plant Pathol..

[46] Summerell B.A. (2019). Resolving Fusarium: Current Status of the Genus. Annu. Rev. Phytopathol..

[47] Hof H. (2020). The Medical Relevance of *Fusarium* spp.. J. Fungi.

[48] Gupta G., Fries B.C. (2010). Variability of phenotypic traits in Cryptococcus varieties and species and the resulting implications for pathogenesis. Futur. Microbiol..

[49] Pal M. (2014). *Cryptococcus gattii*: An emerging global mycotic pathogen of humans and animals. J. Mycopathol. Res..

[50] Da Silva L.L., Moreno H.L.A., Correia H.L.N., Santana M., De Queiroz M.V. (2020). Colletotrichum: Species complexes, lifestyle, and peculiarities of some sources of genetic variability. Appl. Microbiol. Biotechnol..

[51] Perelló A., Gasso M.M.A., Lovisolo M. (2015). Biology and histopathology of *Ustilago filiformis (=U. longissima)*, a causal agent of leaf stripe smut of Glyceria multiflora. J. Plant Prot. Res..

[52] Joshi R. (2018). A Review on Colletotrichum spp. Virulence mechanism against host plant defensive factors. J. Med. Plants Stud..

[53] Valle-Aguirre G., Valle M.G.V.-D., Corona-Rangel M.L., Amora-Lazcano E., Hernández-Lauzardo A.N. (2016). First aeromycological study in an avocado agroecosystem in Mexico. Aerobiologia.

[54] D’Amato G. (2011). Effects of climatic changes and urban air pollution on the rising trends of respiratory allergy and asthma. Multidiscip. Respir. Med..

[55] Kadam K., Karbhal R., Jayaraman V.K., Sawant S., Kulkarni-Kale U. (2017). AllerBase: A comprehensive allergen knowledgebase. Database.

[56] Radauer C., Breiteneder H. (2019). Allergen databases—A critical evaluation. Allergy.

[57] Burton N.C., Grinshpun S.A., Reponen T. (2006). Physical Collection Efficiency of Filter Materials for Bacteria and Viruses. Ann. Occup. Hyg..

[58] Mykytczuk N.C.S., Wilhelm R.C., Whyte L.G. (2012). Planococcus halocryophilus sp. nov., an extreme sub-zero species from high Arctic permafrost. Int. J. Syst. Evol. Microbiol..

[59] Basart S., Pérez C., Cuevas E., Baldasano J.M., Gobbi G.P. (2009). Aerosol characterization in Northern Africa, Northeastern Atlantic, Mediterranean Basin and Middle East from direct-sun AERONET observations. Atmos. Chem. Phys. Discuss..

[60] Perrone M.R., Romano S., Orza J.A.G. (2015). Columnar and ground-level aerosol optical properties: Sensitivity to the transboundary pollution, daily and weekly patterns, and relationships. Environ. Sci. Pollut. Res..

[61] Perrone M.R., Vecchi R., Romano S., Becagli S., Traversi R., Paladini F. (2019). Weekly cycle assessment of PM mass concentrations and sources, and impacts on temperature and wind speed in Southern Italy. Atmos. Res..

[62] Mallet M., Dulac F., Formenti P., Nabat P., Sciare J., Roberts G., Pelon J., Ancellet G., Tanré D., Parol F. (2016). Overview of the Chemistry-Aerosol Mediterranean Experiment/Aerosol Direct Radiative Forcing on the Mediterranean Climate (ChArMEx/ADRIMED) summer 2013 campaign. Atmos. Chem. Phys..

[63] Stein A.F., Draxler R.R., Rolph G.D., Stunder B.J.B., Cohen M.D., Ngan F. (2015). NOAA’s HYSPLIT Atmospheric Transport and Dispersion Modeling System. Bull. Am. Meteorol. Soc..

[64] Haustein K., Pérez C., Baldasano J.M., Jorba O., Basart S., Miller R.L., Janjić Z., Black T., Nickovic S., Todd M.C. (2012). Atmospheric dust modeling from meso to global scales with the online NMMB/BSC-Dust model–Part 2: Experimental campaigns in Northern Africa. Atmos. Chem. Phys. Discuss..

[65] Després V.R., Huffman J.A., Burrows S.M., Hoose C., Safatov A.S., Buryak G., Fröhlich-Nowoisky J., Elbert W., Andreae M.O., Pöschl U. (2012). Primary biological aerosol particles in the atmosphere: A review. Tellus B. Chem. Phys. Meteorol..

[66] White T.J., Bruns T., Lee S., Taylor J., Innis M.A., Gelfand D.H., Sninsky J.J., White T.J. (1990). Amplification and direct sequencing of fungal ribosomal RNA genes for phylogenetics. PCR Protocols: A Guide to Methods and Applications.

[67] Wood D.E., Salzberg S.L. (2014). Kraken: Ultrafast metagenomic sequence classification using exact alignments. Genome Biol..

[68] O’Leary N.A., Wright M.W., Brister J.R., Ciufo S., Haddad D., McVeigh R., Rajput B., Robbertse B., Smith-White B., Ako-Adjei D. (2016). Reference sequence (RefSeq) database at NCBI: Current status, taxonomic expansion, and functional annotation. Nucleic Acids Res..

[69] Shannon C.E. (1948). A mathematical theory of communication. Bell Labs Tech. J..

[70] Simpson E. (1949). Measurement of diversity. Nature.

[71] Haegeman B., Hamelin J., Moriarty J., Neal P., Dushoff J., Weitz J.S. (2013). Robust estimation of microbial diversity in theory and in practice. ISME J..

[72] Chernov T.I., Tkhakakhova A.K., Kutovaya O.V. (2015). Assessment of diversity indices for the characterization of the soil prokaryotic community by metagenomic analysis. Eurasian Soil Sci..

[73] Escobar-Zepeda A., De León A.V.-P., Sanchez-Flores A. (2015). The Road to Metagenomics: From Microbiology to DNA Sequencing Technologies and Bioinformatics. Front. Genet..

[74] Kim B.-R., Shin J., Guevarra R.B., Lee J.H., Kim D.W., Seol K.-H., Lee J.-H., Kim H.B., Isaacson R.E. (2017). Deciphering Diversity Indices for a Better Understanding of Microbial Communities. J. Microbiol. Biotechnol..

[75] Krebs C.J., Krebs C.J. (2014). Species diversity measures. Ecological Methodology.

[76] Schloss P.D., Westcott S.L., Ryabin T., Hall J.R., Hartmann M., Hollister E.B., Lesniewski R.A., Oakley B.B., Parks D.H., Robinson C.J. (2009). Introducing mothur: Open-Source, Platform-Independent, Community-Supported Software for Describing and Comparing Microbial Communities. Appl. Environ. Microbiol..

[77] Magurran A.E. (2004). An index of diversity. Measuring Biological Diversity.

[78] Ricotta C., Podani J. (2017). On some properties of the Bray-Curtis dissimilarity and their ecological meaning. Ecol. Complex..

[79] Hammer O., Harper D.A.T., Ryan P.D. (2001). PAST: Paleontological Statistics Software Package for Education and Data Analysis. Palaeontol. Electron..

[80] Hervé M.R., Nicolè F., Le Cao K.-A. (2018). Multivariate Analysis of Multiple Datasets: A Practical Guide for Chemical Ecology. J. Chem. Ecol..

[81] Legendre P., Anderson M.J. (1999). Distance-Based Redundancy Analysis: Testing Multispecies Responses in Multifactorial Ecological Experiments. Ecol. Monogr..

[82] Jones D.L. (2017). Fathom Toolbox for MATLAB: Software for Multivariate Ecological and Oceanographic Data Analysis.

