# Supplementary material for: Seasonal Variability of the Airborne Eukaryotic Community Structure at a Coastal Site of the Central Mediterranean
Source: Toxins (Basel). 2021 Jul 24;13(8):518. doi: 10.3390/toxins13080518 (PMC8402549; doi:10.3390/toxins13080518)
Supplement: Supplementary file 1 [file toxins-13-00518-s001.zip › toxins-1294531 supplementary figure S1-S9.pdf]

Supplementary Materials: Seasonal Variability of the Airborne Eukaryotic Community Structure at a Coastal Site of the Central Mediterranean

Mattia Fragola, Maria Rita Perrone, Pietro Alifano, Adelfia Talà and Salvatore Romano

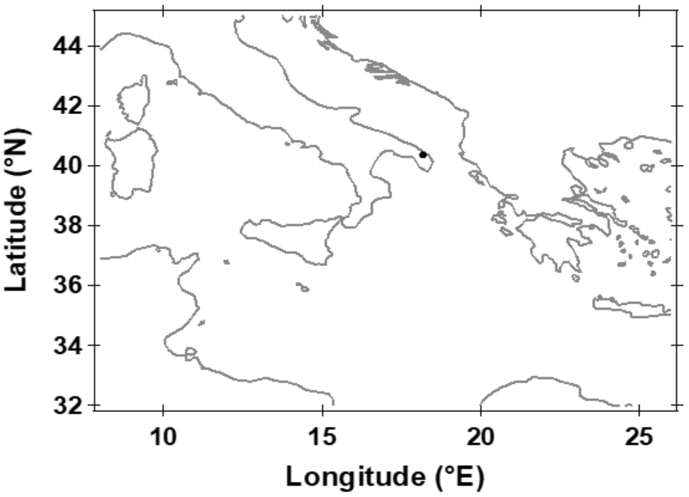

Figure S1. Geographical location of the monitoring site at the Mathematics and Physics Department of the University of Salento in Lecce, Italy (black dot) in the Central Mediterranean Basin.

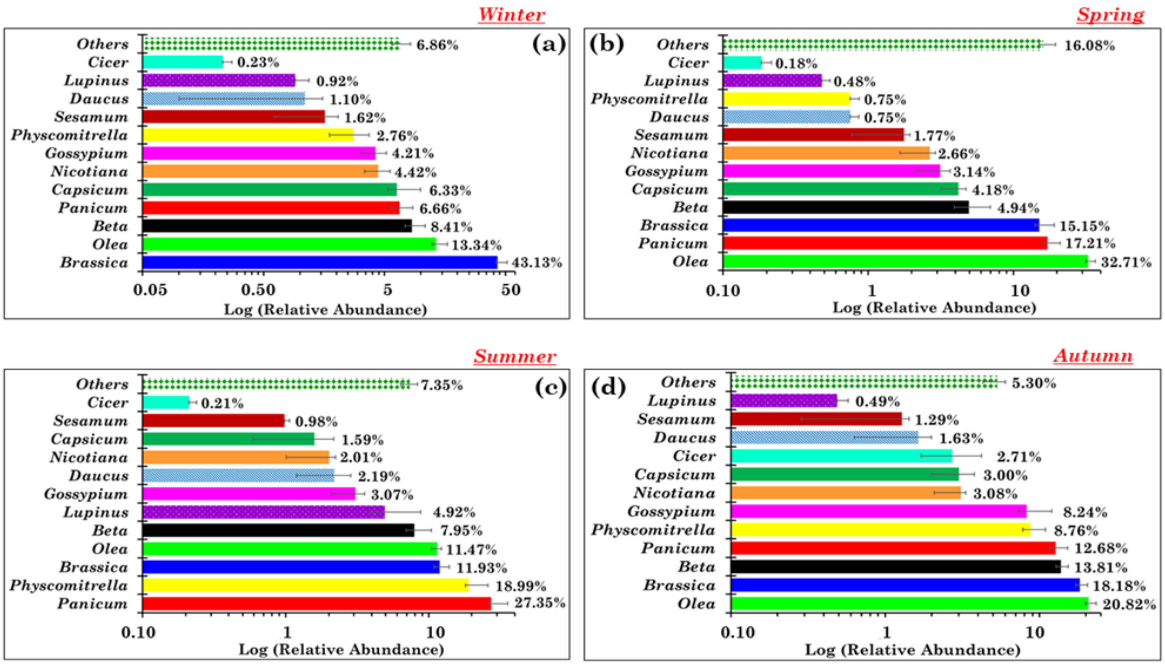

Figure S2. Mean percentage contribution (on a logarithmic scale) of the 12 most abundant and pervasive Streptophyta genera (≥1.17% mean within-sample relative abundance) in (**a**) winter, (**b**) spring, (**c**) summer and (**d**) autumn samples. The error bars represent the standard error of the mean. The <1.17% mean within-sample relative abundance genera, in addition to the not-pervasive high-RA ones, are grouped as *"Others"*.

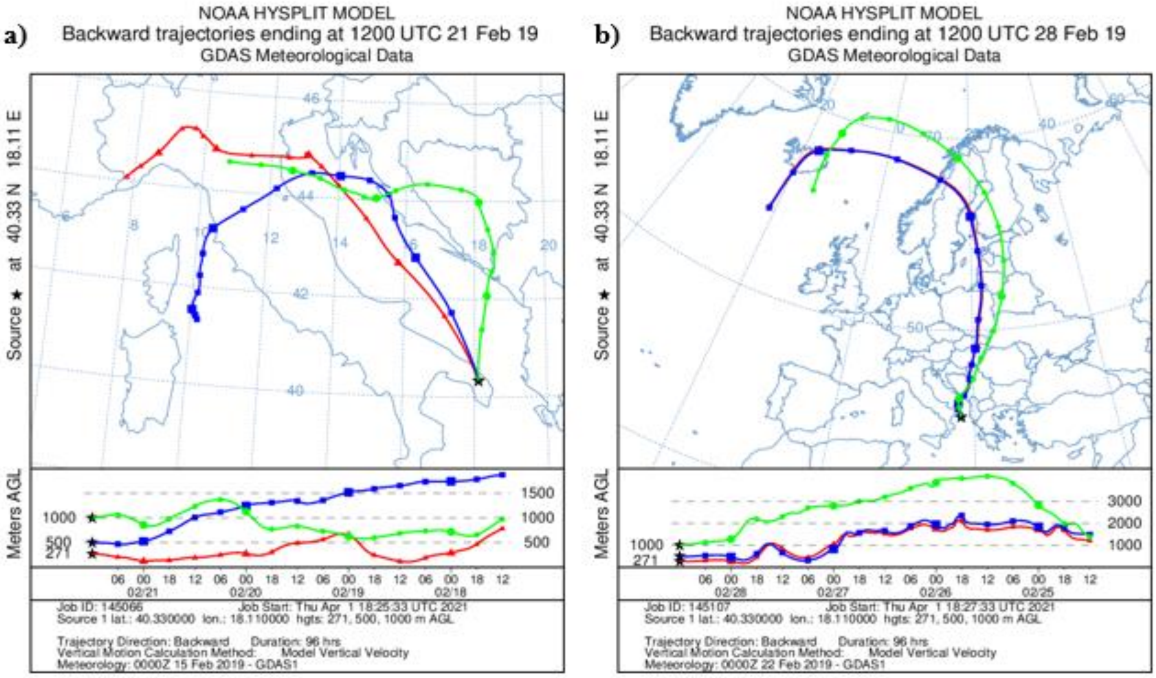

Figure S3. Four-day analytical back-trajectories that reached the study site at 271, 500, and 1000 m AGL, at 12:00 UTC on February (**a**) 21 and (**b**) 28, 2019, when 24-hour samples S7 and S8 were collected, respectively.

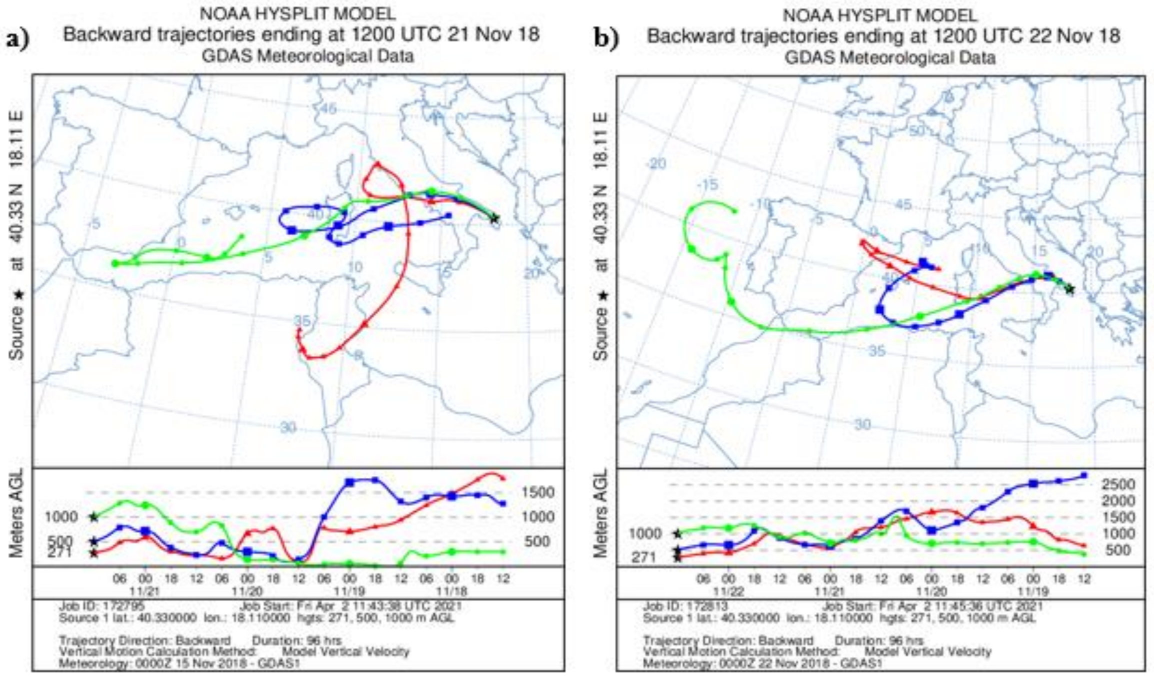

Figure S4. Four-day analytical back-trajectories that reached the study site at 271, 500, and 1000 m AGL, at 12:00 UTC on November (**a**) 21 and (**b**) 22, 2018, when 48-hour samples S28 and S29 were collected, respectively.

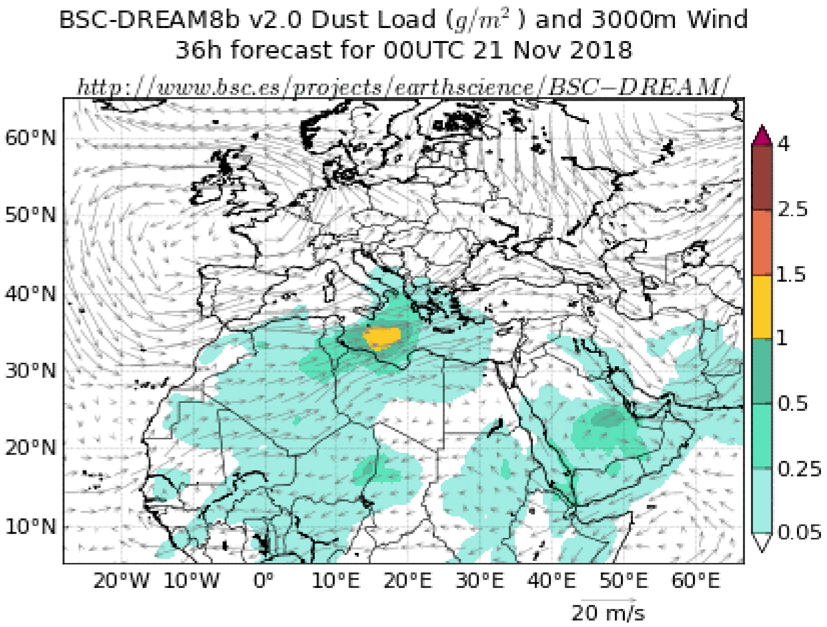

Figure S5. Dust load map from the BSC-DREAM8b model in the Central Mediterranean basin at 00:00 UTC on November 21, 2018, when 48-hour sample S28 was collected.

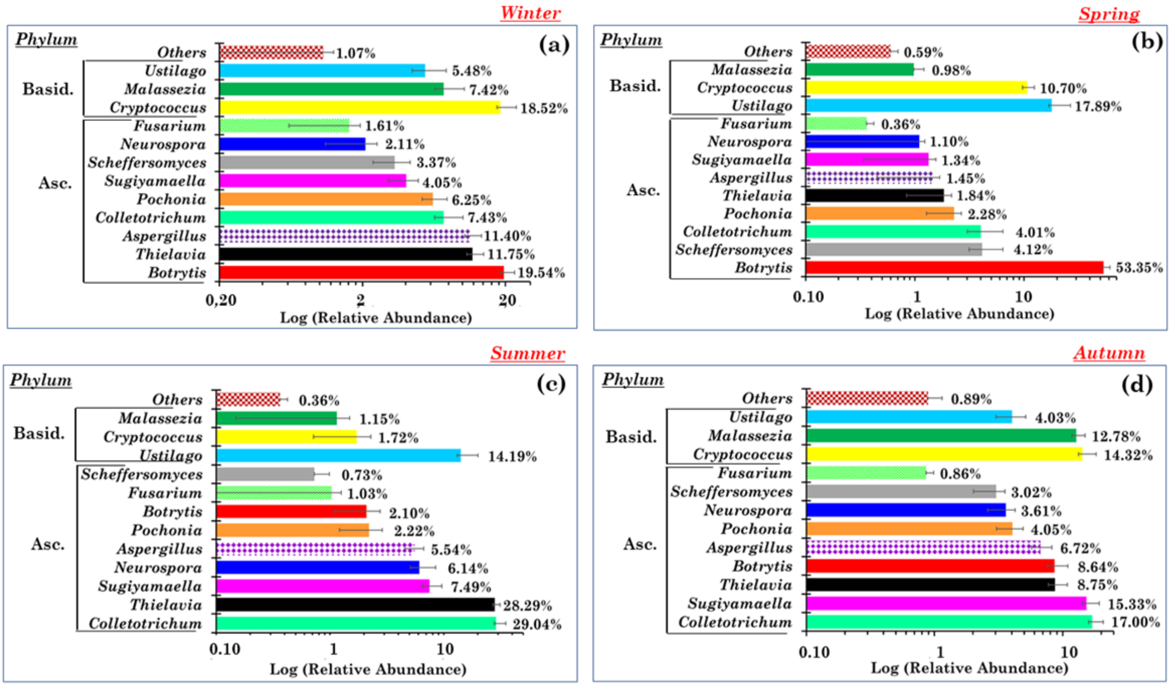

Figure S6. Mean percentage contribution (on a logarithmic scale) of the 12 most abundant and pervasive Ascomycota-Basidiomycota genera (≥0.95% mean within-sample relative abundance) in (**a**) winter, (**b**) spring, (**c**) summer and (**d**) autumn samples. The error bars represent the standard error of the mean. The <0.95% mean within-sample relative abundance genera, in addition to the not-pervasive high-RA ones, are grouped as *"Others"*. Phyla related to each genus are also reported on the left (Basid.: Basidiomycota, Asc.: Ascomycota).

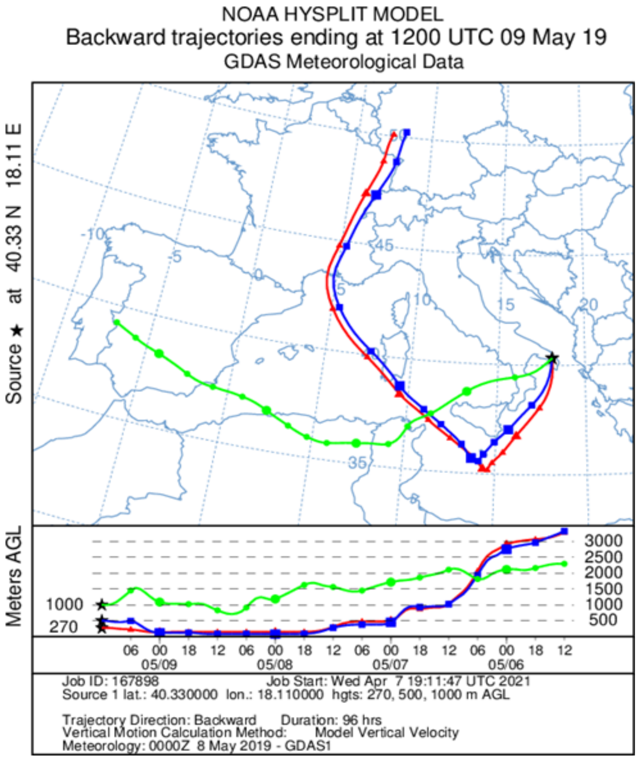

Figure S7. Four-day analytical back-trajectories that reached the study site at 270, 500, and 1000 m AGL, at 12:00 UTC on May 9, 2019, when 24-hour sample S11 was collected.

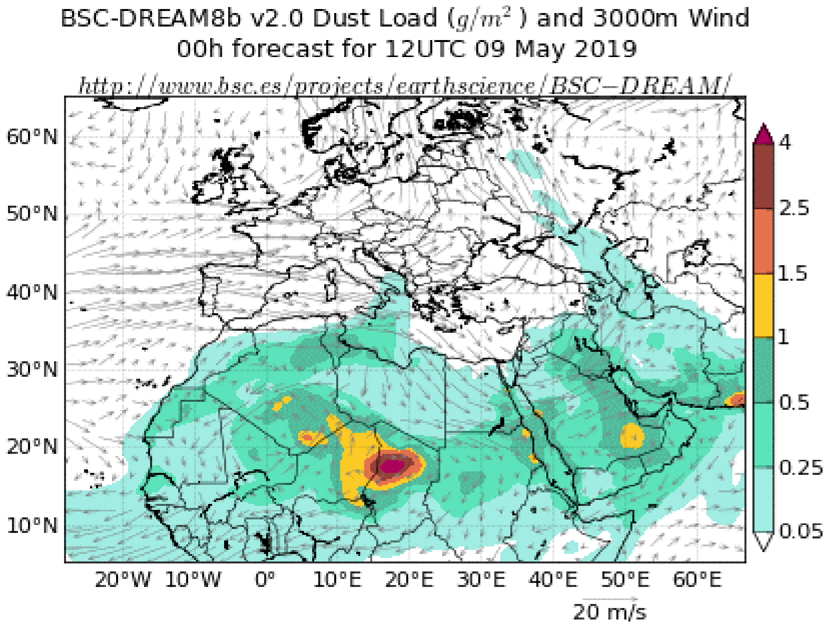

Figure S8. Dust load map from the BSC-DREAM8b model in the Central Mediterranean basin at 12:00 UTC on May 9, 2019, when 24-hour sample S11 was collected.

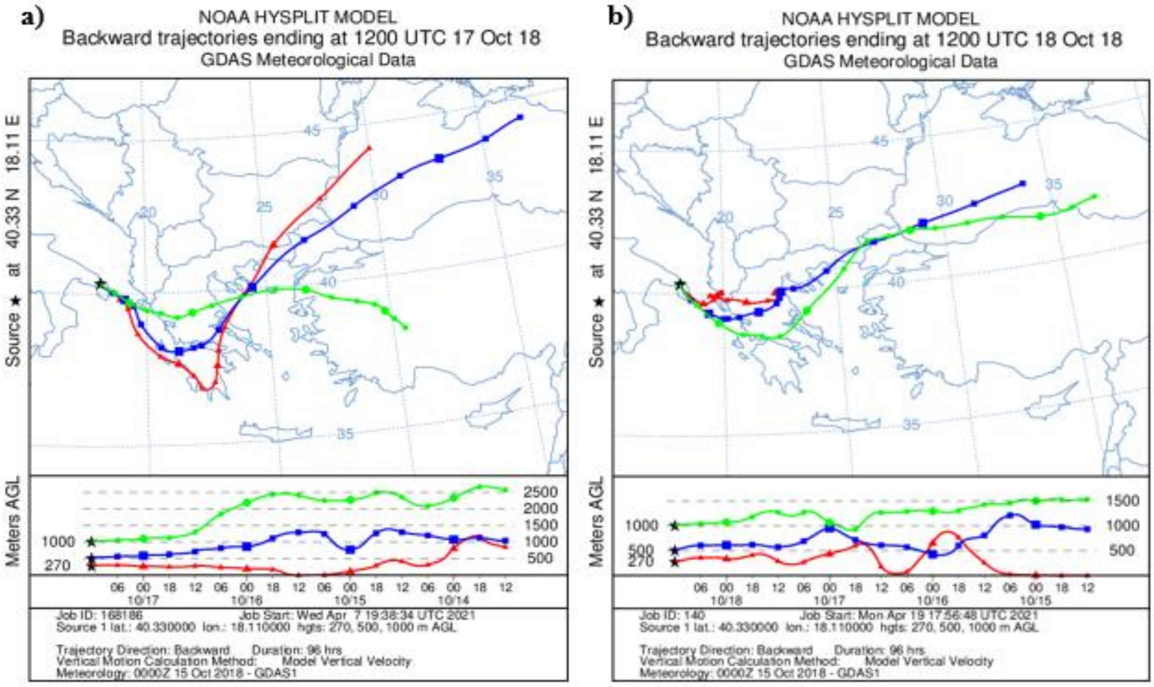

Figure S9. Four-day analytical back-trajectories that reached the study site at 270, 500, and 1000 m AGL, at 12:00 UTC on October (**a**) 17 and (**b**) 18, 2018, when the 48-hour sample Scheme 23. was collected.
